# Hip Osteoarthritis and Physical Workload: Influence of Study Quality on Risk Estimations—A Meta-Analysis of Epidemiological Findings

**DOI:** 10.3390/ijerph16030322

**Published:** 2019-01-24

**Authors:** Yi Sun, Annette Nold, Ulrich Glitsch, Frank Bochmann

**Affiliations:** 1 Unit Applied Epidemiology, Institute for Occupational Safety and Health of the German Social Accident Insurance, 53757 Sankt Augustin, Germany; annette.nold@dguv.de (A.N.); frank.bochmann@dguv.de (F.B.); 2 Unit Musculoskeletal Workload, Institute for Occupational Safety and Health of the German Social Accident Insurance, 53757 Sankt Augustin, Germany; ulrich.glitsch@dguv.de

**Keywords:** osteoarthritis of the hip, coxarthrosis, study quality, meta-analysis, meta-regression analysis, workload, occupational risk

## Abstract

In this paper, we critically evaluate the quality of epidemiological evidence on hip osteoarthritis and workload published so far. The influence of study quality on risk estimations was analyzed in sensitivity meta-analyses and meta-regression analyses. Comprehensive searches for epidemiological studies of hip osteoarthritis and occupational workload were performed in literature databases and current reviews. All studies were assessed on the basis of study design, defined quality scores, and relevant confounders considered. In total, 34 suitable studies were identified for critical evaluation. Of these, 20 are prevalence studies and 14 incidence studies. Strong heterogeneity is observed in study design, quality level, and estimated exposure parameters. A consistent positive association between heavy physical workload and hip osteoarthritis was observed only among the male populations, not among the female populations. In general, cohort studies provided lower effect estimates than cross-sectional and population-based case-control studies. Studies with high quality scores also produced lower effect estimates than studies with low quality scores. Consideration of BMI as a confounder in published studies also yielded lower effect estimates than studies without consideration of BMI as a confounder. Our analyses indicate that high-quality studies of the association between occupational workload and hip osteoarthritis provide lower effect estimates than studies of lower quality.

## 1. Introduction

Osteoarthritis (OA), a degenerative disorder of the joint cartilage and its underlying bone, is the most prevalent joint disease. OA of the hip produces significant morbidity and is a major public health problem throughout the world [1]. Clinical and epidemiological studies of hip OA have revealed a series of etiological factors including both localized factors (such as malformations or joint injuries) and systemic factors (such as being overweight, race, sex, or metabolic diseases) [1]. As a relevant modifying risk factor for hip OA, occupational workload has long been a subject of epidemiological studies. In 2012, we published a systematic review of epidemiological evidence for workload as a risk factor for hip OA [2]. This review indicated that long-term heavy lifting and standing could increase the risk of hip OA. However, the quality difference between the underlying studies may play an important role in determining the size of the estimated effect. It seems that studies with low quality scores tended to report higher effect estimates than studies with higher quality scores [2]. However, this hypothesis is based only on an empirical observation without detailed analysis.

The impact of study quality on the size of effect estimates have been discussed in some methodological papers, which indicates the importance of this issue [3]. An early meta-analysis demonstrated that low quality studies do increase the size of effect estimates of psychotherapy for adult depression [4]. However, the issue is controversially discussed in the literature [3].

The objective in this paper is to critically evaluate the quality of epidemiological evidence by using an update of our previous review [2] and to quantify its possible influences on risk estimations in sensitivity meta-analyses and meta-regression analyses.

## 2. Materials and Methods

### 2.1. Systematic Literature Searches

The meta-analyses of this review are based mainly on the library established for our previous review [2], which includes publications up to 2010. To update the literature, we conducted comprehensive searches in multiple databases, including PubMed, EMBASE, Cochrane Work, and Google Scholar for studies published between January 1, 2010, and December 31, 2017. Several reviews on this topic have been published since 2010 [5,6,7,8,9,10,11,12,13,14]. Comparison with reference lists in reviews completed our extended literature searches and revealed additional studies published before 2010. All titles and abstracts were reviewed by two independent researchers who selected 85 papers for full text reviews. Details of the search strategy are described in File S1. For a clearer view of the available studies, we listed all studies from our updated library: studies considered for critical evaluation and meta-analysis are described and referenced in this publication. Studies excluded from this meta-analysis are listed in Table S1, together with the reasons for their exclusion and the references.

Exclusion criteria were primarily:

- Studies did not address the topic, or address non-idiopathic hip osteoarthritis
- Absence of occupational exposure data
- Studies did not provide 95% CI for effect estimates

Only one of the studies published before 2010 found through cross-referencing met the selection criteria and was included in this meta-analysis [15]. Finally, a total of 33 relevant studies, including the studies of our previous review, were used for this meta-analysis. We followed the Preferred Reporting Items for Systematic Reviews and Meta-Analyses (PRISMA) Statement for reporting all outcomes of our meta-analysis (Table S2).

### 2.2. Classification of Type of Studies for Evidence Comparison

The evidence level of epidemiological studies depends on a series of methodological issues, such as design, sample size, exposure assessment methods, relevant confounders considered, diagnostic criteria, statistical analysis methods used, and so on. For a quality-based comparison of study findings, we divided the evidence published to date into two main groups: prevalence studies (cross-sectional and prevalence case-control studies) and incidence studies. Incidence is a direct measure of risk. In contrast, prevalence often represents a proxy measure of risk, since prevalence depends not only on the risk of diseases, but also on their duration, which is often not readily measurable. Incidence studies therefore generally provide more robust evidences of risk estimation than the prevalence studies. For methodological reasons, a pooling of incidence and prevalence studies is therefore not recommended. According to the hierarchical order in epidemiology, cohort studies (including nested case-control studies) generally provide higher evidence levels than population-based case-control studies. We further divided the incidence studies into a subgroup of cohort studies (including nested case-control studies) and a subgroup of population-based case-control studies.

### 2.3. Classification of Quality Level of Published Studies

Each study identified in this paper was evaluated according to the quality of its case ascertainment (diagnosis criteria) and exposure assessment methods used, as shown in Table 1 and Table 2 respectively. These classification schemas have already been published in our previous review [2].

### 2.4. Statistical Analysis

The summarized effect estimates (relative risk (RR) and odds ratio (OR)) of the relevant studies were quantified via meta-analysis. Two types of exposure parameter were considered in the analysis: heavy lifting (heavy lifting, heavy load handling, and heavy physical work) and farming. Only the highest category of occupational exposures in each study was considered in the meta-analyses. The influence of study quality on the size of effect estimates of occupational workload were assessed by meta-analysis stratified by various type of studies and their quality levels, and additionally by meta-regression analysis.

Meta-analysis was conducted via use of the inverse variance heterogeneity model (IVhet) with the software package Meta XL, version 5.3 (EpiGear International, Sunrise Beach, Australia). I^2^ was used to present the heterogeneity of individual studies within a meta-analysis. Usually, an I^2^ value of 25%, 50%, and 75% was used to represent low, moderate, and high heterogeneity, respectively. High heterogeneity indicates that the individual study findings are inconsistent [16].

Meta-regression analysis was carried out by using random-effects mixed models with the inverse-distance weighted methods with the software package Stata 13. Log OR/RR was used as the dependent variable in the model. Type of study, quality score of exposure assessment and case ascertainment methods, confounders considered in published studies, and sex were used as independent variables in the model.

## 3. Results

**Figure 1:**
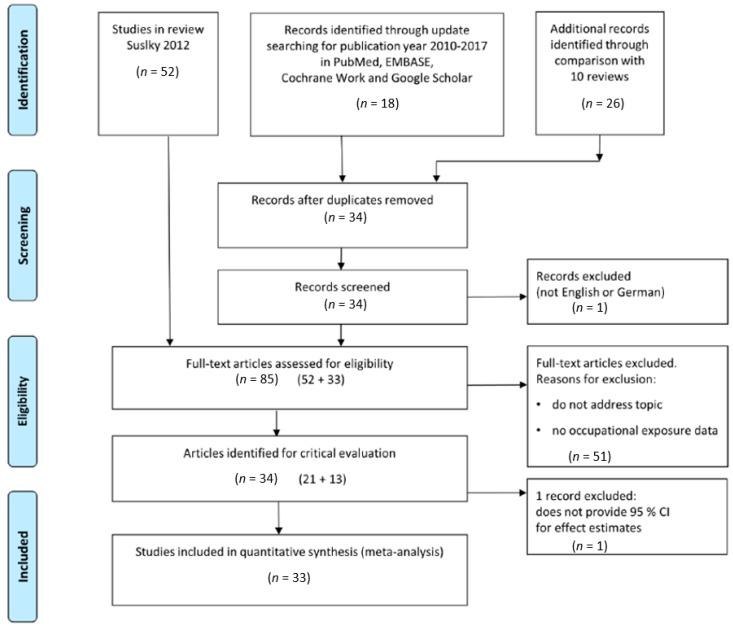
PRISMA (Preferred Reporting Items for Systematic Reviews and Meta-Analyses) flow diagram for the selection of literature for critical evaluation.

We identified 52 relevant articles in the library of our previous review [2] and 33 new studies via the literature searches for the time interval between 2010 and 2017 and by additional searches. Following a full-text review, 21 of the 52 studies published before 2010 and 13 of the 33 new studies met the primary inclusion criteria. This yielded a total of 34 studies that were eligible for critical evaluation. The process of the literature search and selection of studies is summarized in a PRISMA flow diagram as shown in Figure 1.

A description of the study design, sample size, quality level (with consideration for case ascertainment, exposure assessment methods and important confounders), and effect estimates (RR/OR) of these studies is shown in Table 3. Twenty-three of these effect estimates were stated for men, one for women, and 10 for both sexes with adjustment for sex.

Twenty of these studies are prevalence studies (8 cross-sectional and 12 prevalence case-control studies), and 14 are incidence studies (11 cohort and 3 population-based case-control studies). Strong heterogeneity is observed between these studies in their study design, quality level, and estimated exposure parameters. Since one study did not provide a 95% CI for the effect estimate [28], it was not considered in this meta-analysis.

**Figure 2:**
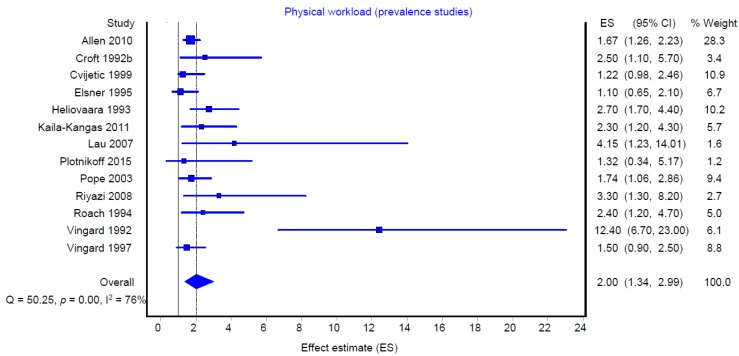
Meta-analysis of heavy physical workload/heavy lifting and the risk of hip osteoarthritis (all prevalence studies, *n* = 13).

**Figure 3:**
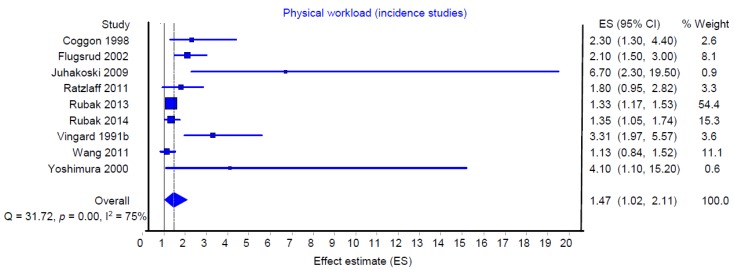
Meta-analysis of heavy physical workload/heavy lifting and the risk of hip osteoarthritis (all incidence studies, *n* = 9).

Figure 2 and Figure 3 show the summarized effect estimates of “heavy lifting” (heavy lifting, heavy load handling and heavy physical work) on hip OA among all prevalence and incidence studies, respectively.

The summarized effect estimate of the prevalence studies was 2.00 (95% CI: 1.34–2.99), and that of the incidence studies was 1.47 (1.02–2.11). Both analyses indicated a strong heterogeneity of individual study findings within the prevalence studies (I^2^ = 76%) and incidence studies (I^2^ = 75%).

**Figure 4:**
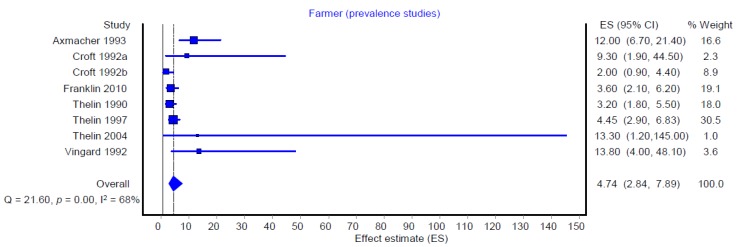
Meta-analysis of the risk of hip osteoarthritis among farmers in prevalence studies (all prevalence studies, *n* = 8).

**Figure 5:**
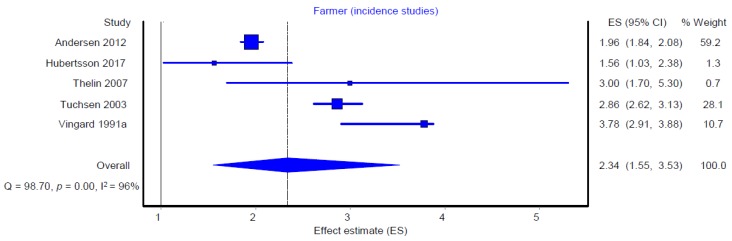
Meta-analysis of the risk of hip osteoarthritis among farmers in cohort studies (all incidence studies, *n* = 5).

The same analyses were also carried out for the exposure parameter of “farming.” The summarized effect estimates of farming for hip OA were 4.74 (95% CI: 2.84–7.89) for the prevalence studies (Figure 4) and 2.34 (95% CI: 1.55–3.53) for the incidence studies (Figure 5, cohort studies only). The analyses also indicate strong heterogeneity between the individual study findings published to date (I^2^ = 68% among prevalence studies and I^2^ = 96% among cohort studies).

In order to assess the influence of study quality on the published epidemiological evidences of the association between heavy occupational workload and hip OA, we carried out a sensitivity meta-analysis for all published studies using different combinations of quality levels and for different types of study, as shown in Table 4.

Independently of the exposure parameters assessed and the type of study, combinations of high quality scores showed lower effect estimates (Table 4). Cohort studies (including nested case-control studies) yielded lower effect estimates than cross-sectional and population-based case-control studies. The heterogeneity of individual study findings decreased with increasing quality level (File S2 contains the forest plots of all meta-analyses carried out in Table 4).

We also conducted the sensitivity meta-analyses separately for men and women. Results for males are summarized in Table 5 and for females in Table 6.

In total, 23 effect estimates were available for men and 13 for women. Among male populations (Table 5), all types of study demonstrated a consistent and statistically significant association between heavy physical workload and hip OA. However, the effect estimates of high-quality prevalence (OR = 2.74, 95% CI: 1.51–4.96) and incidence case-control studies (OR = 2.84, 95% CI: 1.91–4.22) were approximately twice as high as those of cohort studies (OR = 1.33, 95% CI: 1.19–1.50) (Table 5). Among the prevalence studies for males, no influence of study quality on the size of effect estimates was observed. However, among cohort studies, high-quality studies yielded the lowest effect estimates (Table 5). For the exposure parameter of “farmer,” higher-quality studies also yielded much lower effect estimates than those of lower-quality studies (Table 5). Similarly, the effect estimates of prevalence studies (OR = 3.85, 95% CI: 2.06–7.21) are much higher than those of cohort studies (OR = 2.34, 95% CI: 1.54–3.54) (Table 5). Forest plots of all meta-analyses carried out in Table 5 are shown in File S3.

Among female populations, a statistically significant association between heavy physical workload and hip OA was not observed in any type of study (Table 6). The influence of study quality on risk estimations could not therefore be examined among female populations. Female farmers appeared to have an increased risk of hip OA (OR = 1.22, 95% CI: 1.12–1.33) (Table 6). However, only two studies with the same quality level were available. Forest plots of all meta-analyses carried out in Table 6 are shown in File S4.

Table 7 demonstrates the results of meta-regressions of all 22 studies available for “heavy lifting” and 13 studies for “farming.”

Overall, meta-regressions consistently demonstrated that cohort studies yielded lower effect estimates than cross-sectional and population-based case-control studies (Table 7). Studies with higher quality scores (case ascertainment and exposure assessments methods) produced lower effect estimates than studies with lower quality scores (Table 7). Similarly, studies with consideration of BMI as a confounder in the analysis provide lower effect estimates in general than studies without consideration of BMI as confounder. However, consideration of prior injury as a confounder may lead to either an increased or a decreased effect size of occupational workload on hip OA.

## 4. Discussion

The objective of this analysis was not to discuss possible effect estimates for the association between heavy occupational workload and hip OA, but rather to discuss an important methodological issue that may have a strong influence on the interpretation of epidemiological evidences published to date.

The association between heavy occupational workload and hip OA has been reported consistently in the scientific literature, but the size of effect estimates and the specific occupational activities reported as increasing risk vary tremendously between studies. A previous review indicated that studies with low quality scores tended to report higher effect estimates than studies with high quality scores [50]. However, this hypothesis was based only on certain empirical observations, without detailed analysis.

In this analysis, we sought to prove whether this hypothesis was justified in a sensitivity meta-analysis and meta-regression analysis of 33 studies identified in a systematic literature review. Although a number of quality criteria (design, sample size, case ascertainment methods, exposure assessment methods, and important confounders considered) were assessed in this review, not all of them could be considered in the sensitivity meta-analyses and meta-regression analyses, owing to the limited number of studies.

In addition to the differences in design and quality level, consideration of sex in individual studies made a comparison of study findings difficult. For example, of the 34 studies identified, 11 were carried out among male populations [15,20,21,27,36,39,40,42,43,45,46], one among a female population [47], and 22 among mixed-sex populations [17,18,19,22,23,24,25,26,28,29,30,31,32,33,34,35,37,38,39,41,44,48,49]. Of the 22 studies with mixed-sex groups, effect estimates were stated separately for men and women in only 12 studies. In the other 10, effect estimates were only adjusted for sex, and not stratified. This yielded a total of 23 effect estimates for male populations, 13 for female populations, and 13 for populations of both sexes. We decided to focus our analyses primarily on male populations. Therefore, 23 effect estimates stated in Table 3 are for male populations, one for a female population, and 10 for populations of both sexes. The results of sensitivity meta-analyses presented in Table 4 and meta-regression analyses presented in Table 7 were based on the effect estimates stated in Table 3.

To avoid any possible bias due to the sex differences in Table 4, we also carried out the sensitivity meta-analyses separately for men and women and meta-regression analyses adjusted for sex. The findings of our analyses indicated that no association exists between heavy occupational workload and hip OA among the female populations. There was therefore no need to carry out sensitivity meta-analyses among the female populations. Female farmers appeared to have an increased risk of hip OA. However, the influence of study quality on this association could not be examined due to the limited number of only two studies.

Among the male populations, a consistent association between heavy physical workload and hip OA was observed for all types of study. However, studies of high-evidence design (cohort and nested case-control studies) showed only half the effect estimates of the studies of low-evidence design (cross-sectional and population-based case-control studies). This phenomenon was demonstrated consistently for the two exposure parameters (heavy physical workload and farmer) estimated, and in both meta-analyses and meta-regressions. The combination of high quality scores appeared to have less influence on the size of effect estimates (for exposure parameter “heavy physical workload”) in the prevalence studies (Table 5). However, among cohort studies, studies with high quality scores still produced lower effect estimates than studies with lower quality scores. In this context, we must emphasize that one cohort study [29] with lower quality scores was not considered in the analyses presented in Table 5, since it provided effect estimates only for both sexes together. The effect estimate for men can be expected to be even higher. Had this study been considered in Table 5, the trend would be much clearer. For the exposure parameter “farmer,” studies with higher quality scores showed lower effect estimates than studies with lower quality scores; this was true for all types of studies. The same findings could also be demonstrated in the meta-regression analyses among all studies adjusted for sex (Table 7). Interestingly we found in the meta-regressions that adjustment of BMI as a confounder in published studies yielded lower effect estimates in general than studies without consideration of BMI as a confounder. This indicates a positive association between occupational workload and BMI among heavy physical workers in published studies. In contrast, prior injury could either be positively or negatively associated with occupational load or the type hip OA (unilateral und bilateral). Therefore, adjustment of prior injury as a confounder in published studies may either positively or negatively influence the effect estimates of occupational workload.

Exposure assessment is the most important and difficult part in occupational epidemiological studies, particularly for exposure–response estimation. Although heavy occupational workload is of major concern in this analysis, the use of different exposure parameters, jobs, or activities in individual studies makes summarization of the effect estimates difficult. Besides heavy lifting and load handling, effect estimates were also stated in some studies for work walking, work standing, stair climbing, crawling, and so on. Since for most exposure parameters and jobs, the number of studies was too small to permit detailed analysis, we limited the sensitivity meta-analyses and meta-regressions to only two exposure parameters, namely “heavy lifting” (heavy lifting, heavy load handling and heavy physical work) and “farming.” These two exposure parameters were selected solely owing to the relatively large number of published studies in which they are included. The results of the sensitivity meta-analyses and meta-regressions performed with these two exposure parameters demonstrated the consistent finding that high-evidence design studies (cohort and nested case-control studies) produced lower effect estimates than low-evidence design studies (cross-sectional and population-based case-control studies). Studies with high quality scores (case ascertainment and exposure assessment methods) also report lower effect estimates than studies with low quality scores.

In the present meta-analyses, we found the heterogeneities of many individual studies to be very high (I^2^ > 50%). This suggests systematic differences between the individual studies. One reason could be the different ways of defining exposure parameters and the use of a reference group in the analysis. In this review, we defined five quality levels for exposure assessment methods. The best exposure assessment score we found among the studies reaches only level 3, which underlines the need for further improvement in exposure assessment methods in well-designed epidemiological studies (cohort and nested case-control studies).

## 5. Conclusions

Overall, our analyses indicate that high-quality studies on the association between heavy occupational workload and hip osteoarthritis generally yield lower effect estimates than studies of poor quality. Due to the high heterogeneity of individual findings published (even among the well-designed studies) and limited number of high quality studies, a conclusive statement on the effect size of the association between heavy occupational workload and hip osteoarthritis should be made with caution. For future research, we see a strong need for better designed studies with large sample sizes, objective exposure assessment methods (active biomechanical measurement for jobs combined with an objective job-exposure-matrix), and valid case ascertainment and sufficient control of relevant confounders. Regulatory decision-making on the management of work related hip osteoarthritis should be based mainly on evidence of well-designed, high-quality studies.

## Figures and Tables

**Table 1:** Definition of level of evidence for diagnostic evaluation of studies on osteoarthritis of the hip (score 1–3) [2].

Diagnosis Criteria	Diagnostic Quality Score * (Evidence Level)
Anamnesis/questionnaire: hip pain without clinical check	1
Hip pain and clinical reduction of movement without radiographic features or Radiographic features without clinical examination, without THR (total hip replacement)	2
Hip pain with clinical reduction of movement and clearly defined radiographic features (joint space narrowing or Kellgren-Lawrence-score grade 2 and above or comparable criteria) or Diagnosis with indication for THR (total hip replacement)	3

* Score 1—low quality; score 3—high quality.

**Table 2:** Definition of quality for exposure assessment of studies on osteoarthritis of the hip (score 1–5) [2].

Exposure Assessment	Exposure Quality Score *
Profession, job title, classification of occupation	1
Qualitative specification of exposure in different work activities (lifting, climbing stairs, sitting)	2
Quantitative specification of exposure in different work activities/physical strains with information on intensity (e.g., load weight steps) and duration	3
Quantitative specification of exposure (as above) with additional plausibility check (e.g., information on daily work output or special controls through video analysis)	4
Quantitative, measured exposure with quantitative assessment or modeling of hip joint strain	5

* Score 1—low quality; score 5—high quality.

**Table 3:** Epidemiological evidence of heavy occupational workload and the risk of hip osteoarthritis.

Study	Design	Outcome Assessment	Study Population		Quality Score of		Confounders Considered/Controlled				Exposure Parameter Estimated	RR/OR ** (95% CI)
			Sample Size	Age (Years)	Exposure Assessment	Hip OA * Ascertainment	Age	Sex	BMI	Prior Injury		
Allen 2010 [17]	Cross-sectional	Prevalence	2729	>45	2	3	✓	✓	✓	✓	Heavy lifting	1.67 (1.26–2.23)
Andersen 2012 [18]	Cohort	Incidence	2.1 million	25–59	1	3	✓	✓		✓	Farmer	1.96 (1.84–2.08)
Axmacher 1993 [15]	Cross-sectional	Prevalence	565 farmers	40–64	1	2	✓	✓			Farmer	12 (6.7–21.4)
Coggon 1998 [19]	Case-control	Incidence	611 cases, 611 controls	45–91	3	3	✓	✓	✓	✓	Heavy lifting	2.3 (1.3–4.4)
Croft 1992a [20]	Cross-sectional	Prevalence	167 farmers, 83 office staff	60–76	1	2	✓	✓			Farmer	9.3 (1.9–44.5)
Croft 1992b [21]	Case-control	Prevalence	245 cases, 294 controls	60–75	3	2	✓	✓			Heavy lifting	2.5 (1.1–5.7)
Cvijetic 1999 [22]	Cross-sectional	Prevalence	593	>45	2	3	✓	✓	✓		Heavy physical	1.22 (0.98–2.46)
Elsner 1995 [23]	Case-control	Prevalence	220 cases, 198 controls	Median ca. 50	1	3	✓	✓			Heavy lifting	1.1 (0.65–2.10)
Flugsrud 2002 [24]	Cohort	Incidence	50,034	Mean 55	2	3	✓	✓	✓		Heavy physical	2.10 (1.50–3.00)
Franklin 2010 [25]	Case-control	Prevalence	1408 cases, 1082 controls	≥60	1	3	✓	✓	✓		Farmer	3.6 (2.1–6.2)
Heliovaara 1993 [26]	Cross-sectional	Prevalence	7217	≥30	2	2	✓	✓	✓	✓	Heavy physical	2.7 (1.7–4.4)
Hubertsson 2017 [27]	Cohort	Incidence	165,179	40–70	1	3	✓	✓			Farmer	1.56 (1.03–2.38)
Jacobsen 2004a [28]	Cross-sectional	Prevalence	4151	22–93	3	2	✓	✓	✓		Heavy lifting	1.0
Juhakoski 2009 [29]	Cohort	Incidence	840	30–72	2	2	✓	✓	✓	✓	Heavy physical	6.7 (2.3–19.5)
Kaila-Kangas 2011 [30]	Cross-sectional	Prevalence	6556	30–97	3	2	✓	✓	✓	✓	Handling load	2.3 (1.2–4.3)
Lau 2007 [31]	Case-control	Prevalence	138 cases, 414 controls	not given	3	3	✓	✓	✓	✓	Heavy lifting	4,15 (1.23–14.0)
Plotnikoff 2015 [32]	Cross-sectional	Prevalence	4733	≥18	1	3	✓	✓			Heavy physical	1.32 (0.34–5.17)
Pope 2003 [33]	Case-control	Prevalence	352 cases, 3002 controls	18–85	3	1	✓	✓			Handling load	1.74 (1.06–2.86)
Ratzlaff 2011 [34]	Cohort	Incidence	2918	45–85	2	3	✓	✓	✓	✓	Heavy physical	1.80 (0.95–2.82)
Riyazi 2008 [35]	Case-control	Prevalence	93 cases, 345 controls	40–79	1	3	✓	✓			Heavy physical	3.3 (1.3–8.2)
Roach 1994 [36]	Case-control	Prevalence	99 cases, 233 controls	Mean 68	3	3	✓	✓	✓	✓	Heavy physical	2.4 (1.2–4.7)
Rubak 2013 [37]	Cohort	Incidence	1.9 million	31–71	3	3	✓	✓			Heavy physical	1.33 (1.17–1.53)
Rubak 2014 [38]	Case-control nested	Incidence	1776 cases, 1776 controls	41–69	3	3	✓	✓	✓	✓	Heavy physical	1.35 (1.05–1.74)
Thelin 1990 [39]	Case-control	Prevalence	105 cases, 222 controls	55–70	1	3	✓	✓			Farmer	3.2 (1.8–5.5)
Thelin 1997 [40]	Case-control	Prevalence	216 cases, 479 controls	<70	2	2	✓	✓			Farmer	4.45 (2.90–6.83)
Thelin 2004 [41]	Case-control	Prevalence	369 cases, 369 controls	40–71	2	3	✓	✓		✓	Farmer	13.3 (1.2–145.0)
Thelin 2007 [42]	Cohort	Incidence	3437	40–59	1	2	✓				Farmer	3.0 (1.7–5.3)
Tuchsen 2003 [43]	Cohort	Incidence	Not given	20–59	1	3	✓	✓			Farmer	2.86 (2.62–3.13)
Vingard 1991a [44]	Cohort	Incidence	250,217	Not given	1	3	✓	✓			Farmer	3.78 (2.91–3.88)
Vingard 1991b [45]	Case-control	Incidence	239 cases, 302 controls	50–70	3	3	✓	✓	✓	✓	Heavy lifting	3.31 (1.97–5.57)
Vingard 1992 [46]	Case-control	Prevalence	140 cases, 298 controls	50–69	1	3	✓	✓			Heavy physical	12.4 (6.7-23.0)
Vingard 1997 [47]	Case-control	Prevalence	230 cases, 273 controls	50–70	3	3	✓	✓	✓		Heavy lifting	1.5 (0.9–2.5)
Wang 2011 [48]	Cohort	Incidence	39,023	27–75	1	3	✓	✓	✓		Heavy physical	1.13 (0.84–1.52)
Yoshimura 2000 [49]	Case-control	Incidence	114 cases, 114 controls	≥45	3	3	✓	✓	✓	✓	Heavy lifting	4.1 (1.1–15.2)

* OA: Osteoarthritis ** RR = Relative Risk; OR = Odds Ratio.

**Table 4:** Results of meta-analysis depending on the type and quality level of published studies.

Exposure Parameters Estimated	Type of Studies	Exposure Score	Diagnostic Score	Number of Studies	Overall ES * (95% CI)	Heterogeneity of Studies (I^2^)
Heavy physical workload/heavy lifting	Prevalence studies	All studies	All studies	13	2.00 (1.34–2.99)	76%
		1–2	1–2	1	2.70 (1.70–4.40)	-
		All Studies	=3	9	1.92 (1.02–3.59)	83%
		≥3	All studies	6	1.98 (1.53–2.58)	0%
		≥3	=3	3	1.94 (1.17–3.20)	30%
	Incidence Studies					
	Case-control	≥3	=3	3	2.93 (2.00–4.28)	0%
	Cohort	All studies	All studies	6	1.40 (1.03–1.89)	71%
		1–2	1–2	1	6.70 (2.30–19.50)	-
		All Studies	=3	5	1.38 (1.12–1.69)	54%
		≥3	=3	2	1.33 (1.19–1.50)	0%
Farmer	Prevalence studies	1–2	All studies	8	4.74 (2.84–7.89)	68%
		1–2	1–2	4	5.38 (2.23–12.97)	79%
		1–2	=3	4	3.97 (2.17–7.26)	46%
	Incidence studies					
	Cohort	1–2	All studies	5	2.34 (1.55–3.53)	96%
		1–2	1–2	1	3.00 (1.70–5.30)	-
		1–2	=3	4	2.34 (1.54–3.54)	97%

* ES: effect estimate.

**Table 5:** Results of meta-analysis depending on the type and quality level of published studies for males.

Exposure Parameters Estimated	Type of Studies	Exposure Score	Diagnostic Score	Number of Studies	Overall ES * (95% CI)	Heterogeneity of Studies (I^2^)
Heavy physical workload/heavy lifting	Prevalence studies	All studies	All studies	8	2.25 (1.13–4.47)	84%
		1–2	1–2	0	-	-
		All Studies	=3	6	2.21 (0.85–5.80)	89%
		≥3	All studies	4	2.52 (1.71–3.70)	0%
		≥3	=3	2	2.74 (1.51–4.96)	0%
	Incidence studies					
	Case-control	≥3	=3	2	2.84 (1.91–4.22)	0%
	Cohort	All studies	All studies	3	1.40 (1.07–1.82)	66%
		1–2	1–2	0	-	-
		All Studies	=3	3	1.40 (1.07–1.82)	66%
		≥3	=3	2	1.33 (1.19–1.50)	0%
Farmer	Prevalence studies	1–2	All studies	7	4.69 (2.78–7.89)	71%
		1–2	1–2	4	5.38 (2.23–12.97)	79%
		1–2	=3	3	3.85 (2.06–7.21)	56%
	Incidence studies					
	Cohort	1–2	All studies	5	2.34 (1.56–3.53)	96%
		1–2	1–2	1	3.00 (1.70–5.30)	-
		1–2	=3	4	2.34 (1.54–3.54)	97%

* ES: effect estimate.

**Table 6:** Results of meta-analysis depending on the type and quality level of published studies for females.

Exposure Parameters Estimated	Type of Studies	Exposure Score	Diagnostic Score	Number of Studies	Overall ES * (95% CI)	Heterogeneity of Studies (I^2^)
Heavy physical workload/heavy lifting	Prevalence studies	All studies	All studies	4	1.69 (1.04–2.75)	47%
		1–2	1–2	0	-	-
		All Studies	=3	3	2.02 (1.10–3.70)	42%
		≥3	All studies	3	1.69 (0.97–2.94)	65%
		≥3	=3	2	2.03 (0.94–4.34)	71%
	Incidence studies					
	Case-control	≥3	=3	1	0.80 (0.40–1.50)	-
	Cohort	All studies	All studies	3	1.06 (0.67–1.69)	77%
		1–2	1–2	0	-	-
		All Studies	=3	3	1.06 (0.67–1.69)	77%
		≥3	=3	2	1.01 (0.89–1.15)	0%
Farmer	Prevalence studies	1–2	All studies	1	0.62 (0.36–1.00)	-
		1–2	1–2	0	-	-
		1–2	=3	1	0.62 (0.36–1.00)	-
	Incidence studies					
	Cohort	1–2	All studies	2	1.22 (1.12–1.33)	0%
		1–2	1–2	0	-	-
		1–2	=3	2	1.22 (1.12–1.33)	0%

* ES: effect estimate.

**Table 7:** Results of meta-regressions of all published studies.

Exposure Parameters Estimated	Independent Parameters in Meta-Regression Model *	β-Values of log (OR/RR) **	Standard Error (SE) of β-Values
Heavy physical workload/heavy lifting (*n* = 22 studies)	Cohort study (yes vs. no)	−0.208	0.301
	Quality score of exposure assessment	−0.183	0.210
	Quality score of case assessment	−0.121	0.305
	BMI as confounder (yes vs. no)	−0.320	0.416
	Prior injury as confounder (yes vs. no)	0.565	0.368
Farmer (*n* =13 studies)	Cohort study (yes vs. no)	−0.877	0.375
	Quality score of exposure assessment	−0.762	0.323
	Quality score of case assessment	−0.450	0.390
	BMI as confounder (yes vs. no)	−0.503	0.587
	Prior injury as confounder (yes vs. no)	−0.234	0.448

* All parameters were considered in the same model; ** Sex was adjusted in the meta-regression models.

## Supplementary Materials

The following are available online at http://www.mdpi.com/1660-4601/16/3/322/s1: File S1: Literature search strategy, File S2: Forest plots of all meta-analyses carried out for Table 4 (all studies), File S3: Forest plots of all meta-analyses carried out for Table 5 (males), File S4: Forest plots of all meta-analyses carried out for Table 6 (females), Table S1: Excluded studies, reasons for exclusion and references, Table S2: PRISMA checklist.
