# Supplementary material for: Hip Osteoarthritis and Physical Workload: Influence of Study Quality on Risk Estimations—A Meta-Analysis of Epidemiological Findings
Source: Int J Environ Res Public Health. 2019 Jan 24;16(3):322. doi: 10.3390/ijerph16030322 (PMC6388382; doi:10.3390/ijerph16030322)
Supplement: Supplementary file 1 [file ijerph-16-00322-s001.zip › File S1.pdf]

File S1. Literature search strategy

To improve precision and recall, test searches were conducted. Tests included synonym controls, check and comparison of MESH terms and terms in EMBASE basic index. Searches were limited to English and German language articles.

Literature with publication date 2010 - 2017 was searched.

PubMed:

17.5.2018

#1 Search "Osteoarthritis, Hip"[Mesh]

#2 Search occupational?

#3 Search lifting or carrying

#4 Search farmer? or farming

#5 Search #2 OR #3 OR #4

#6 Search #1 AND #5

#7 Search ("2010/01/01"[Date - Publication] : "2017/12/31"[Date - Publication])

#8 Search #6 AND #7

N hits = 34

EMBASE:

7.5.2018, Host STN

L1 S HIP OSTEOARTHRITIS

L2 S COXARTHROSIS

L3 S COXARTHROSES

L4 S L1 OR L2 OR L3

L5 S INDUSTRIAL MEDICINE

L6 S OCCUPATION?

L7 S L5 OR L6

L8 S L4 AND L7

L9 S L8 AND 2010-2017/PY

L10 S L9 NOT THERAPY

L11 S L10 NOT SURGERY

N hits = 53

Cochrane Work:

26.4.2018, http://work.cochrane.org/

Hand searching

N = 0

Google Scholar:

2.-5.5.2018, https://scholar.google.com/

Hand searching was conducted with the following terms in different combinations:

Hip osteoarthritis, coxarthrosis, occupation, work, lifting, dose-response, study.

All hits were studies already known.
