# Supplementary material for: Hip Osteoarthritis and Physical Workload: Influence of Study Quality on Risk Estimations—A Meta-Analysis of Epidemiological Findings
Source: Int J Environ Res Public Health. 2019 Jan 24;16(3):322. doi: 10.3390/ijerph16030322 (PMC6388382; doi:10.3390/ijerph16030322)

File S2. Forest plots of all meta-analyses carried out for Table 4 (all studies)

Heavy lifting/load handling and hip osteoarthritis (see Table 4)

a. All prevalence studies, n=13

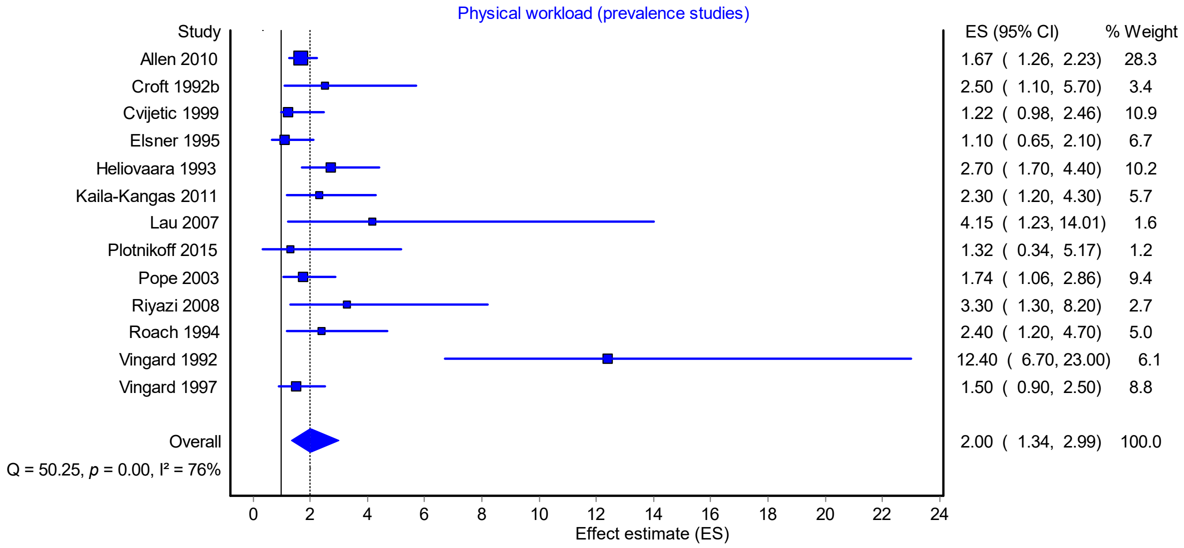

b. Prevalence studies, ExpoScore=all, DiagScore=3, n=9

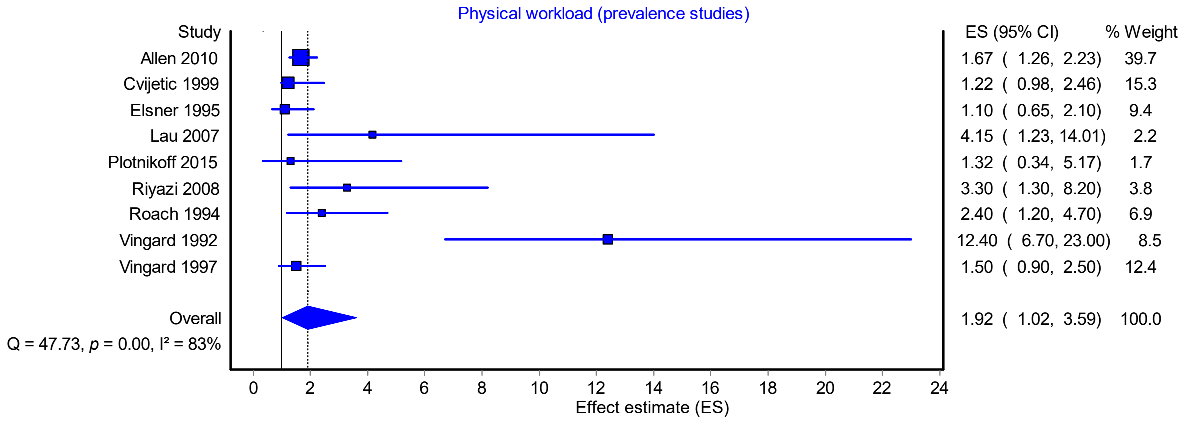

c. Prevalence studies, ExpoScore≥3, DiagScore=all, n=6)

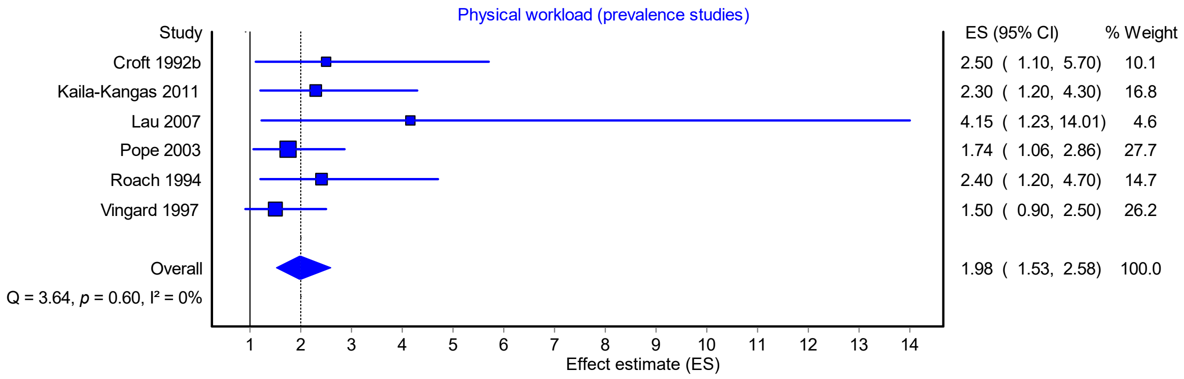

d. Prevalence studies, ExpoScore≥3, DiagScore=3, n=3)

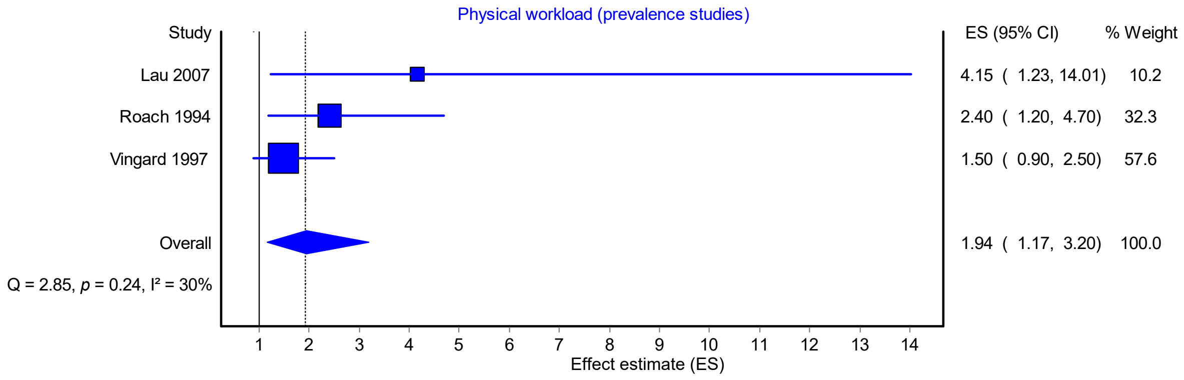

e. All case-control studies, n=3

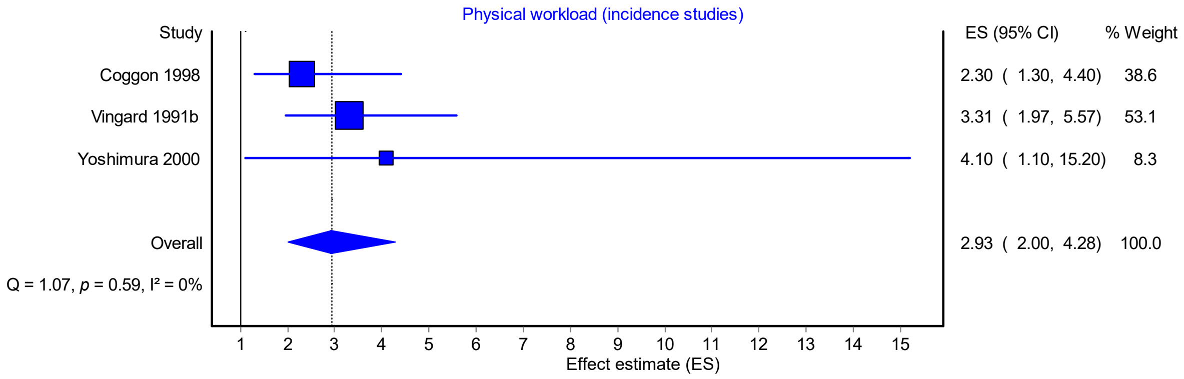

f. All cohort studies, n=6

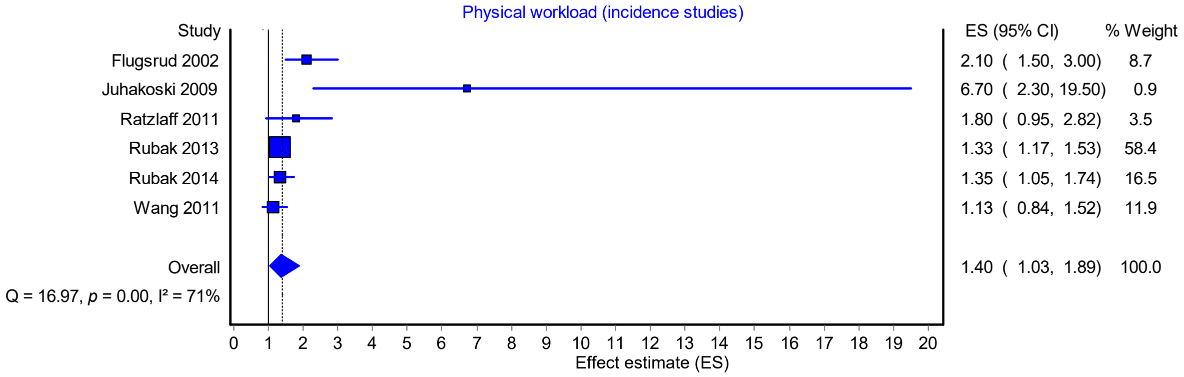

g. Cohort studies, ExpoScore=all, DiagScore=3, n=5

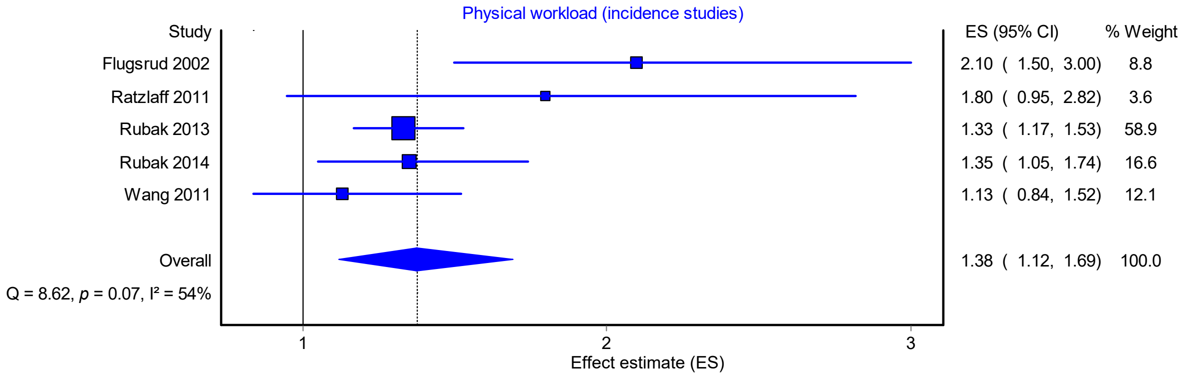

h. Cohort studies, ExpoScore≥3, DiagScore=3, n=2

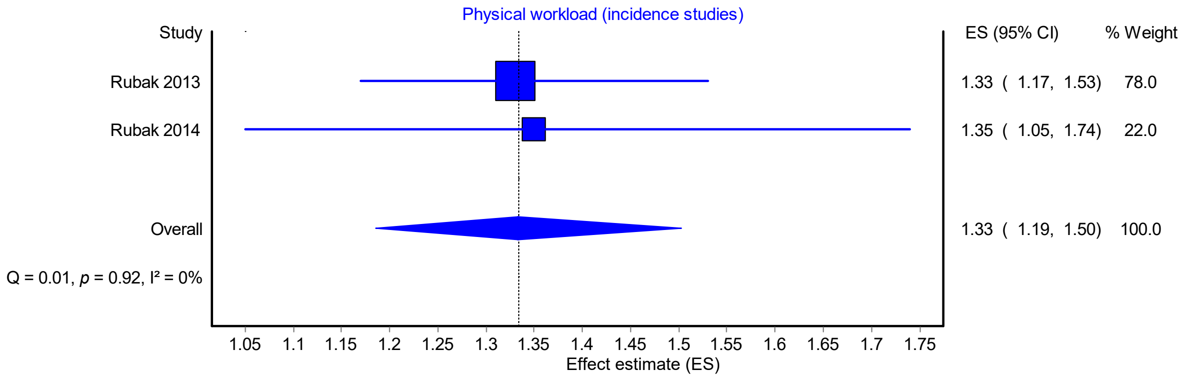

Farming and hip osteoarthritis (see Table 4)

a. All prevalence studies, n=8

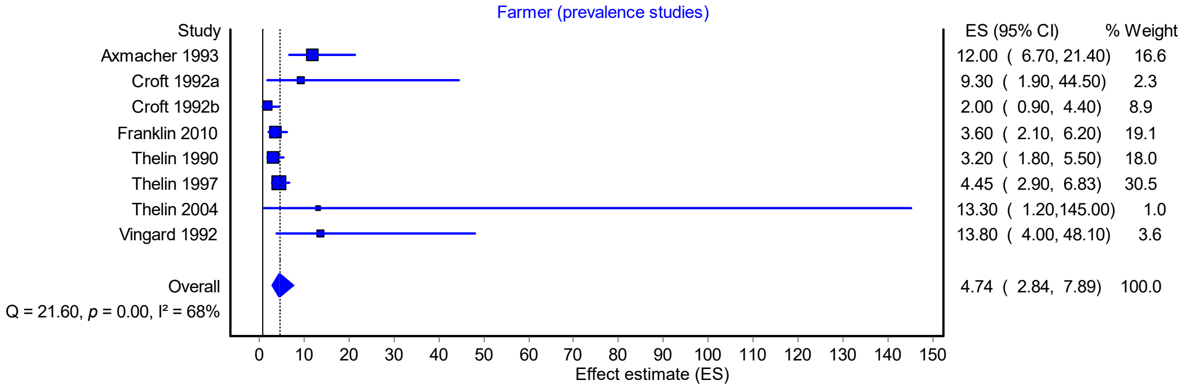

b. Prevalence studies, ExpoScore=1-2, DiagScore=1-2, n=4

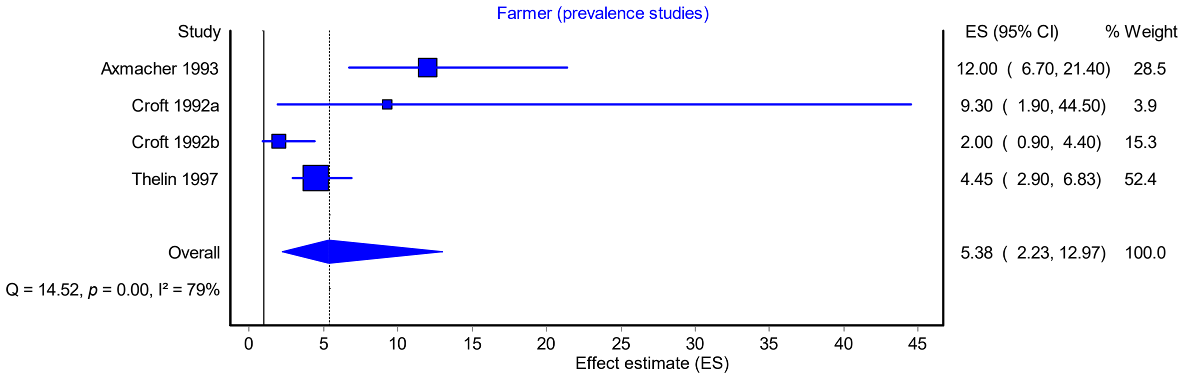

c. Prevalence studies, ExpoScore=1-2, DiagScore=3, n=4

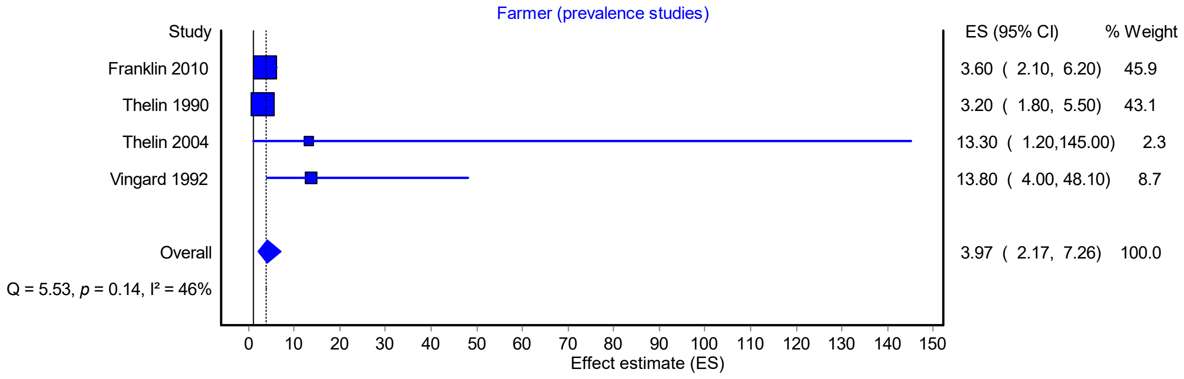

d. All cohort studies, n=5

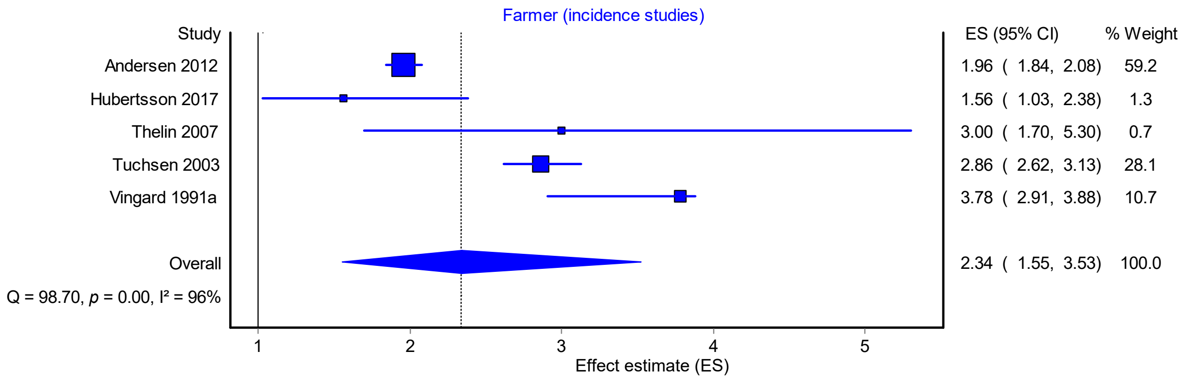

e. Cohort studies, DiagScore=3, n=4

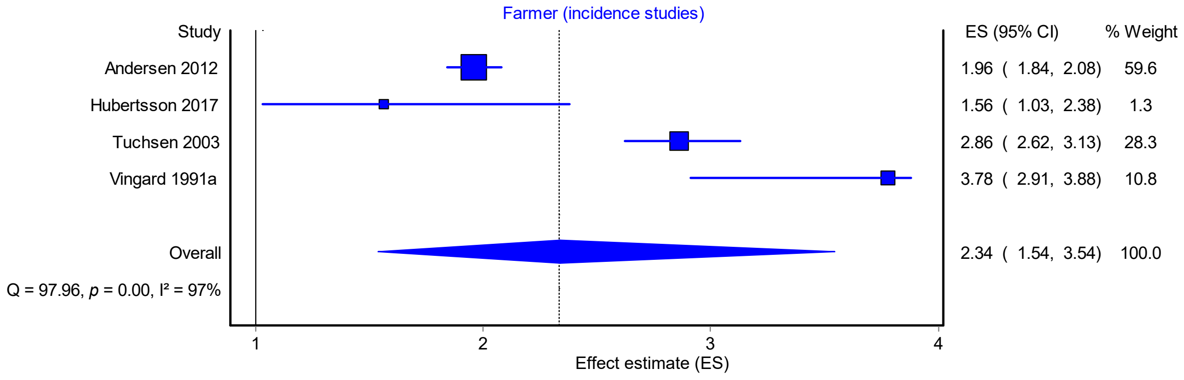

Supplement: Supplementary file 1 [file ijerph-16-00322-s001.zip › File S2.pdf]
