# Supplementary material for: Hip Osteoarthritis and Physical Workload: Influence of Study Quality on Risk Estimations—A Meta-Analysis of Epidemiological Findings
Source: Int J Environ Res Public Health. 2019 Jan 24;16(3):322. doi: 10.3390/ijerph16030322 (PMC6388382; doi:10.3390/ijerph16030322)

File S3. Forest plots of all meta-analyses carried out for Table 5 (males)

Heavy lifting/load handling and hip osteoarthritis among males (see Table 5)

a. All prevalence studies, male, n=8

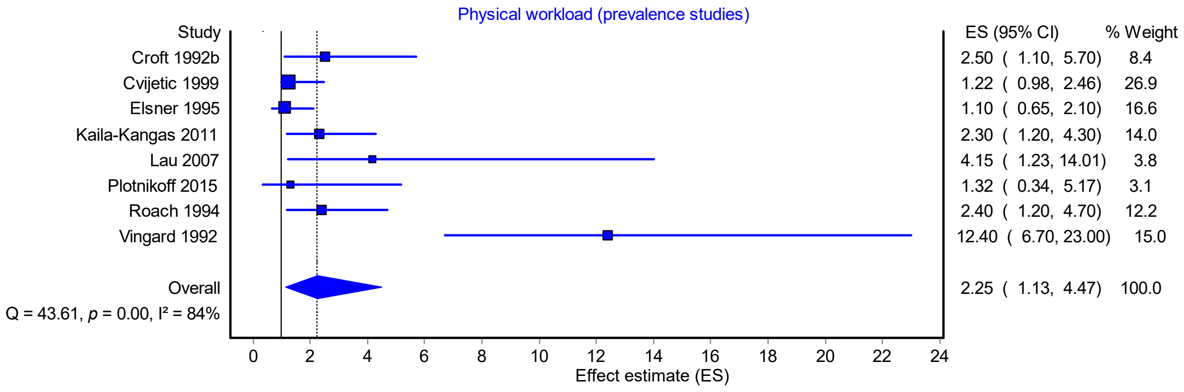

b. Prevalence studies, male, ExpoScore=all, DiagScore=3, n=6

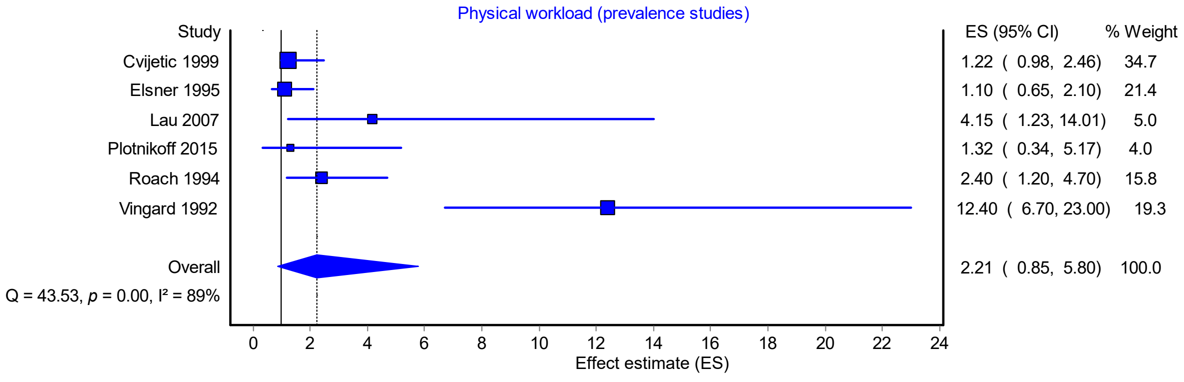

c. Prevalence studies, male, ExpoScore≥3, DiagScore=all, n=4

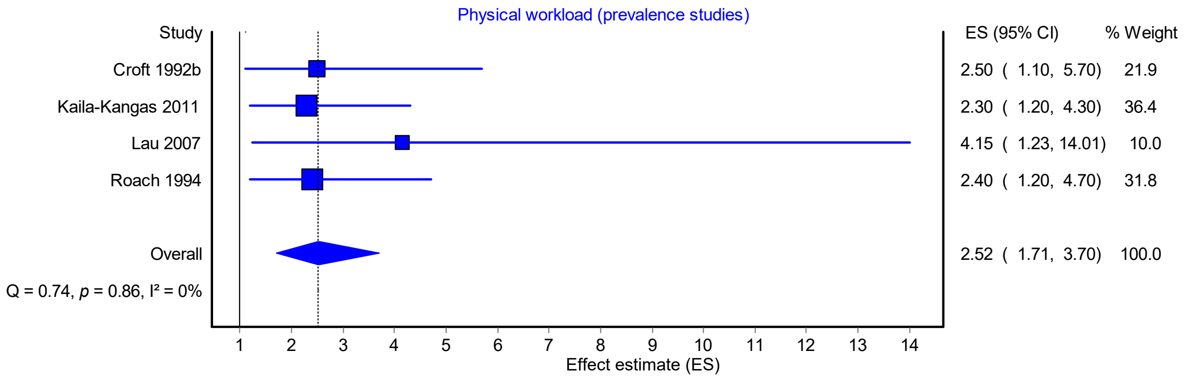

d. Prevalence studies, male, ExpoScore≥3, DiagScore=3, n=2)

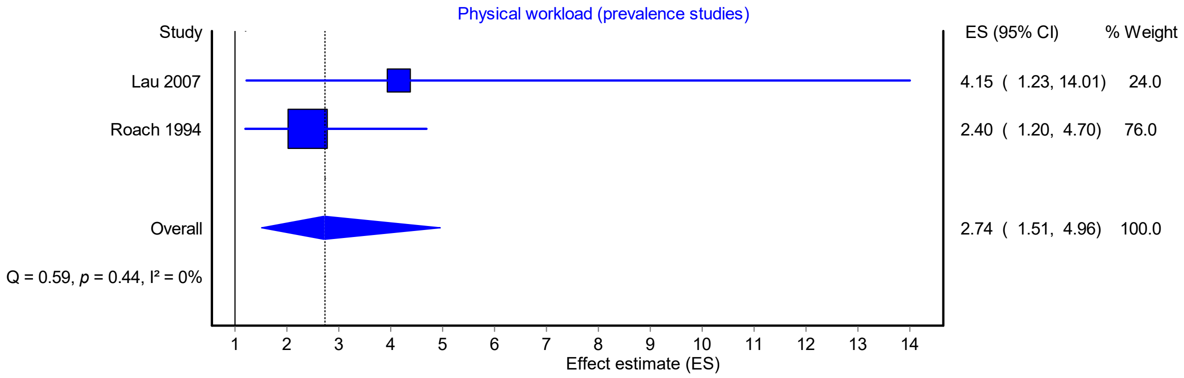

e. All case-control studies, male, n=2

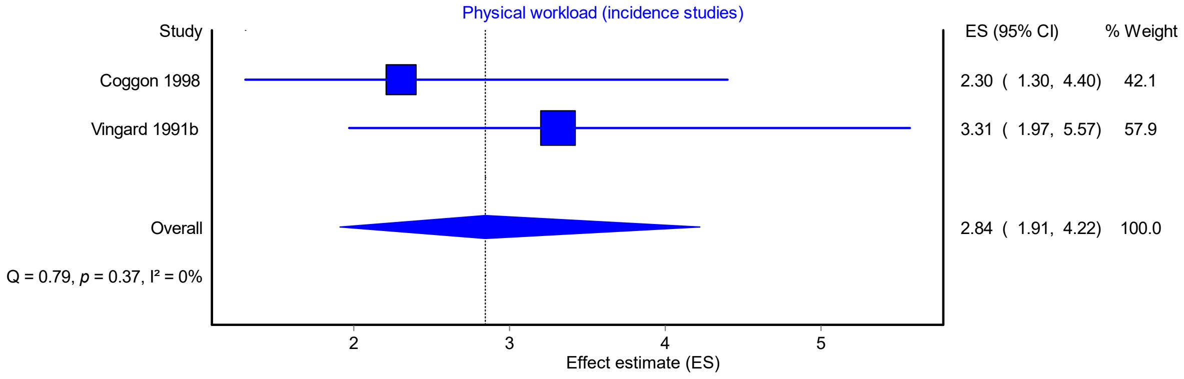

f. All cohort studies, male, n=3

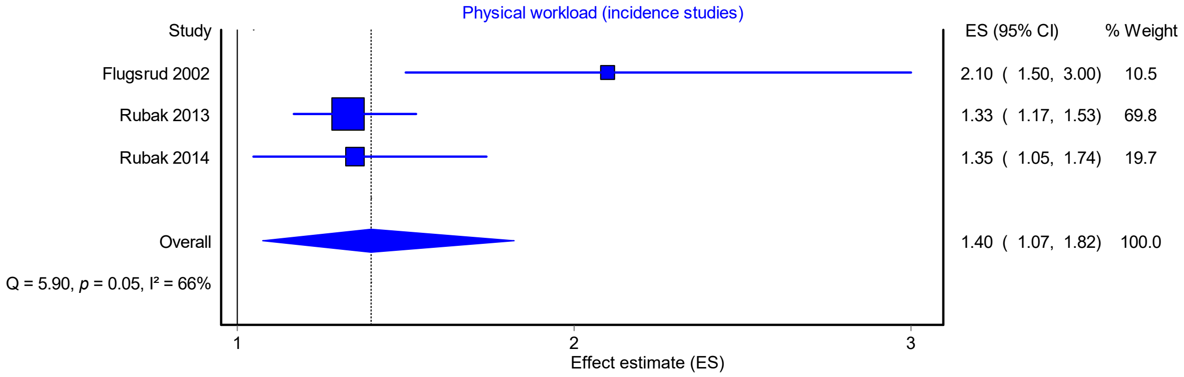

g. Cohort studies, male, ExpoScore≥3, DiagScore=3, n=2

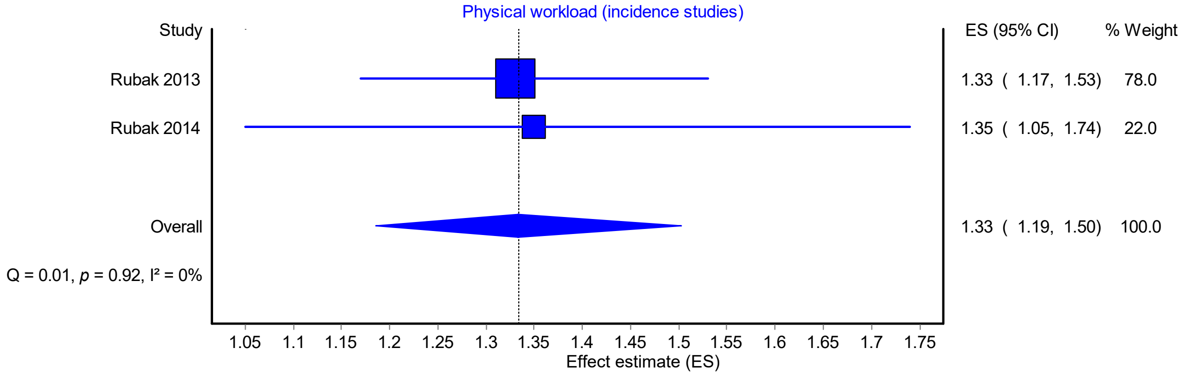

Farming and hip osteoarthritis among males (see Table 5)

a. All prevalence studies, male, n=7

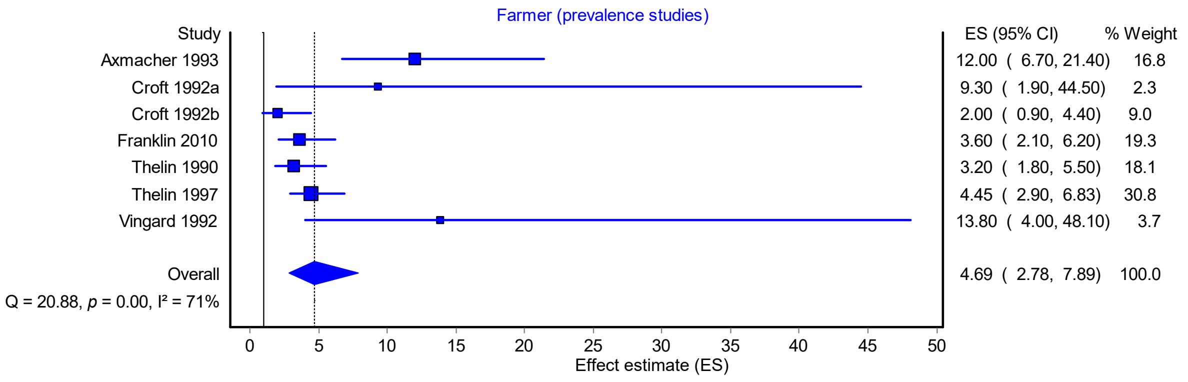

b. Prevalence studies, male, ExpoScore=1-2, DiagScore=1-2, n=4

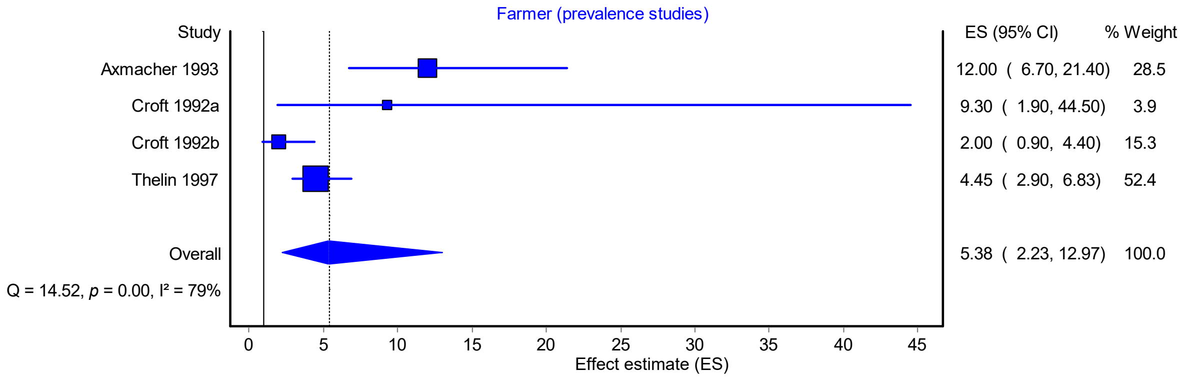

c. Prevalence studies, male, ExpoScore=1-2, DiagScore=3, n=3

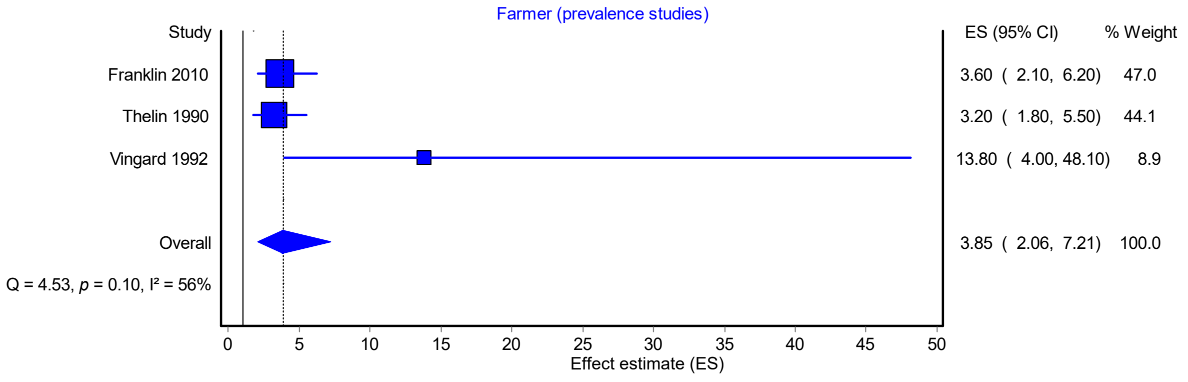

d. All cohort studies, male, n=5

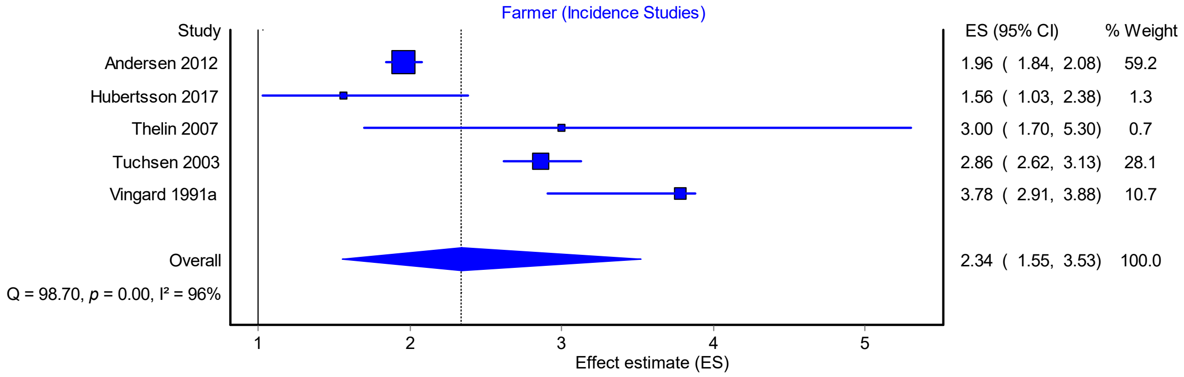

e. Cohort studies, DiagScore=3, n=4

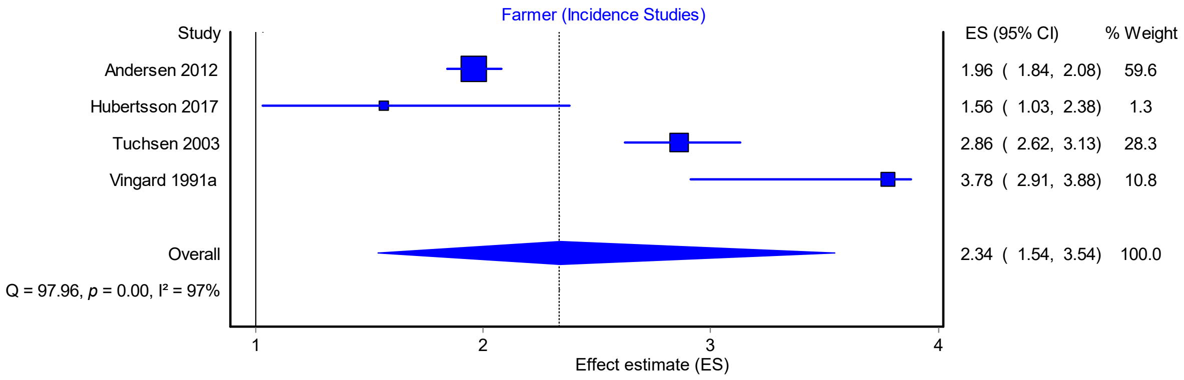

Supplement: Supplementary file 1 [file ijerph-16-00322-s001.zip › File S3.pdf]
