# Supplementary material for: Hip Osteoarthritis and Physical Workload: Influence of Study Quality on Risk Estimations—A Meta-Analysis of Epidemiological Findings
Source: Int J Environ Res Public Health. 2019 Jan 24;16(3):322. doi: 10.3390/ijerph16030322 (PMC6388382; doi:10.3390/ijerph16030322)

File S4. Forest plots of all meta-analyses carried out for Table 6 (females)

Heavy lifting/load handling and hip osteoarthritis among females (see Table 6)

a. All prevalence studies, female, n=4

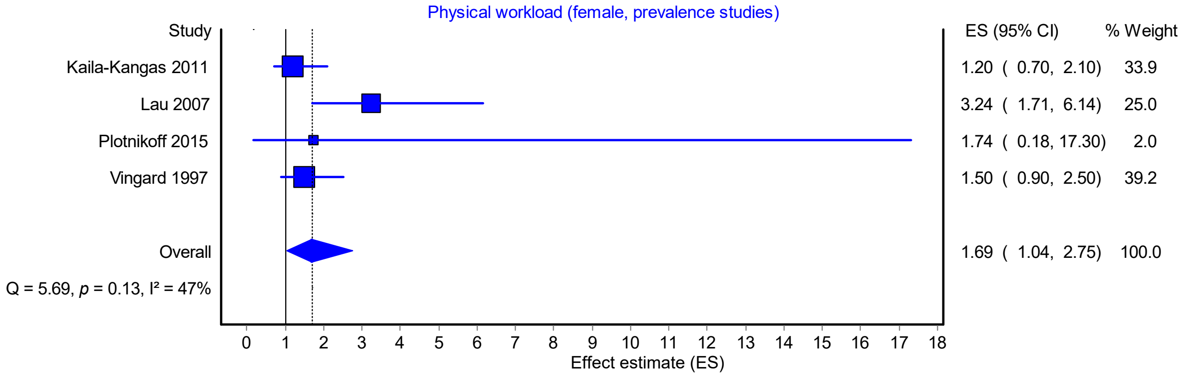

b. All prevalence studies, female, ExpoScore=all, DiagScore=3, n=3

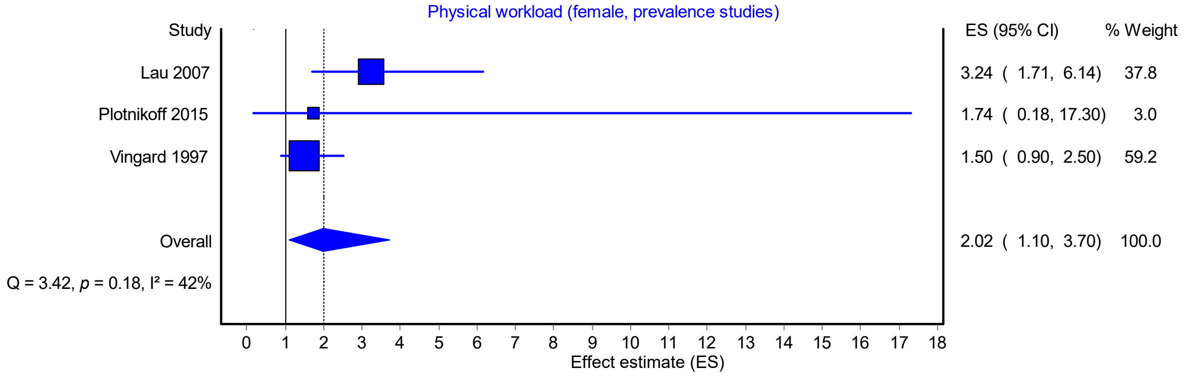

c. All prevalence studies, female, ExpoScore≥3, DiagScore=all, n=3

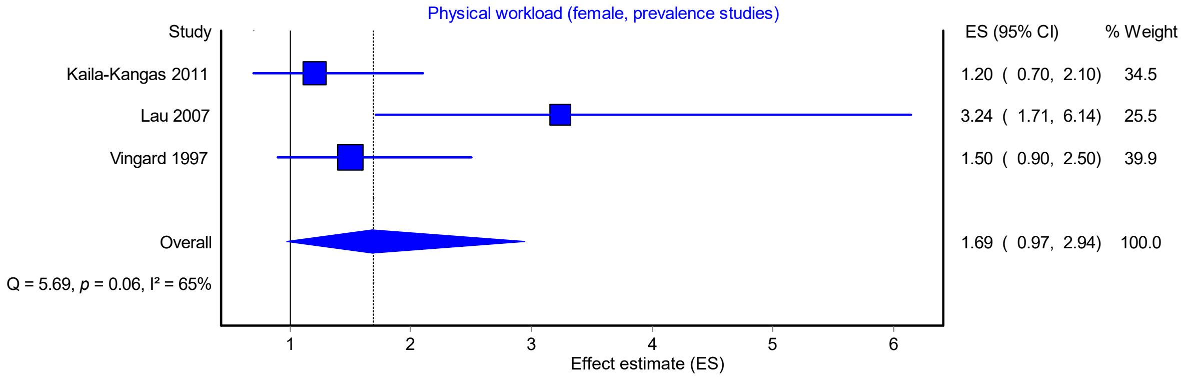

d. All prevalence studies, female, ExpoScore≥3, DiagScore=3, n=2

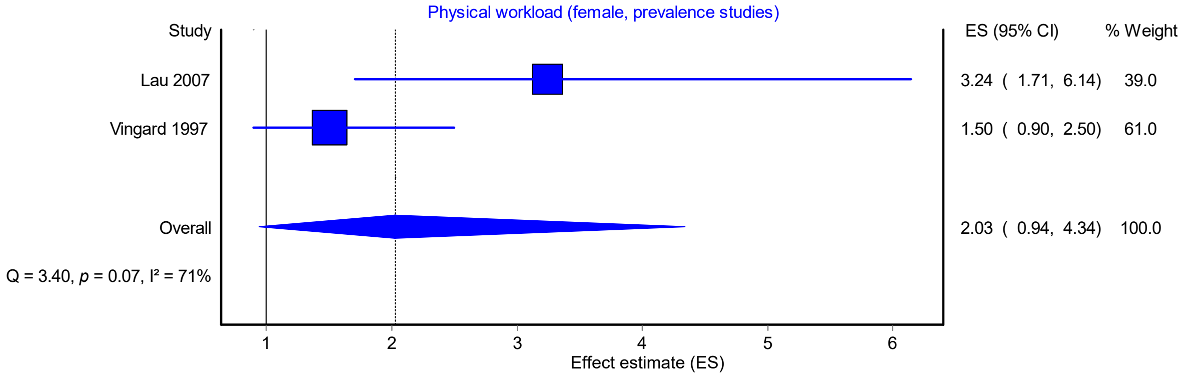

e. All cohort studies, female, n=3

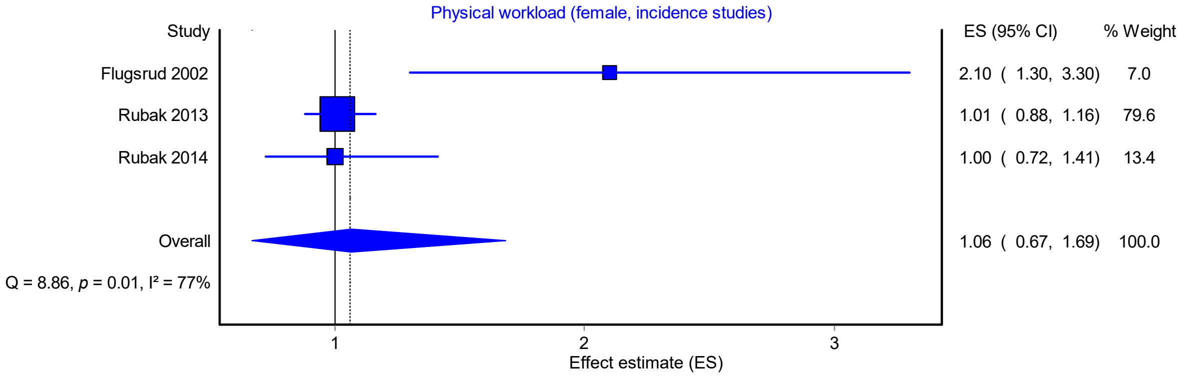

f. Cohort studies, female, ExpoScore=all, DiagScore=3, n=3

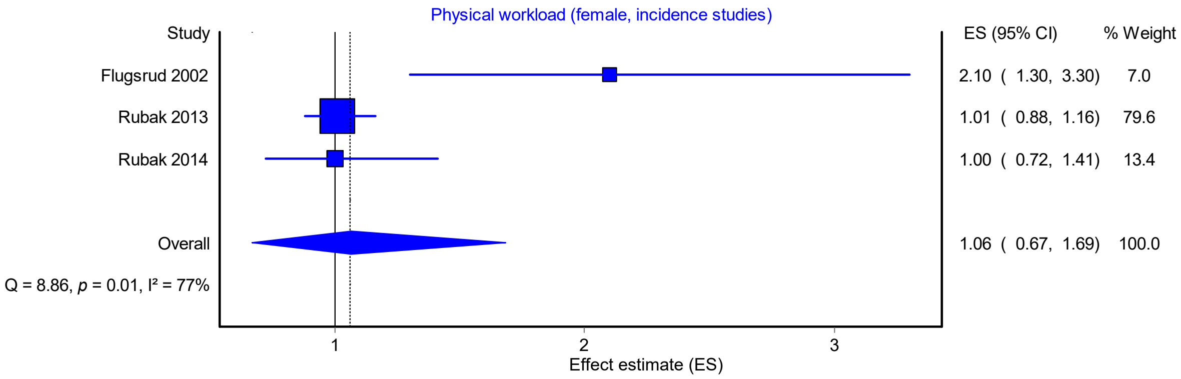

g. Cohort studies, female, ExpoScore≥3, DiagScore=3, n=2

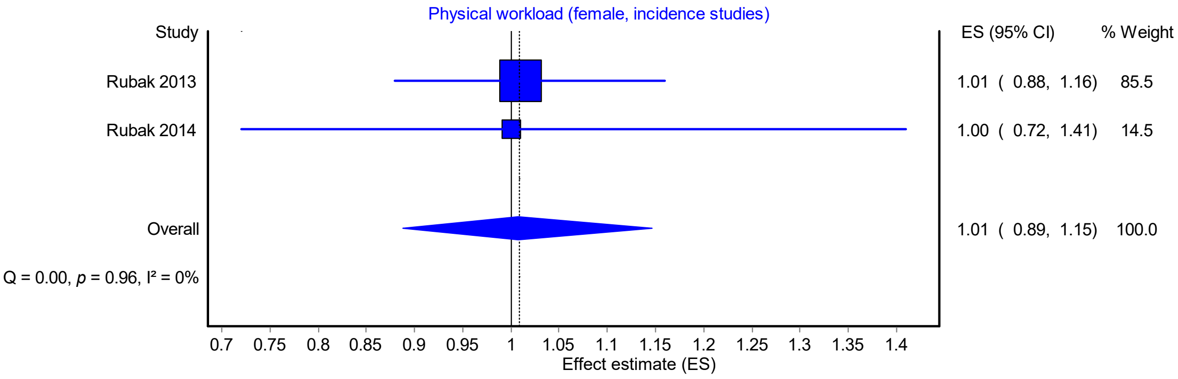

Farming and hip osteoarthritis among females (see Table 6)

a. All cohort studies, female

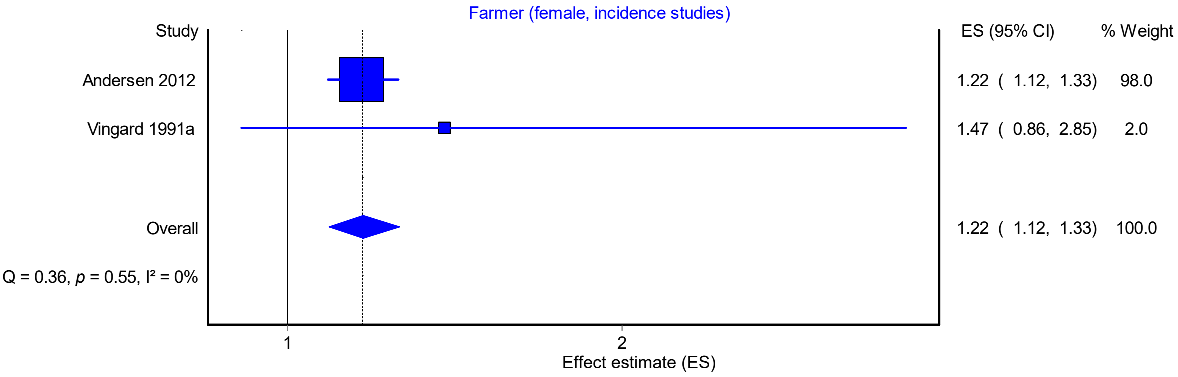

Supplement: Supplementary file 1 [file ijerph-16-00322-s001.zip › File S4.pdf]
