# Supplementary material for: Hip Osteoarthritis and Physical Workload: Influence of Study Quality on Risk Estimations—A Meta-Analysis of Epidemiological Findings
Source: Int J Environ Res Public Health. 2019 Jan 24;16(3):322. doi: 10.3390/ijerph16030322 (PMC6388382; doi:10.3390/ijerph16030322)
Supplement: Supplementary file 1 [file ijerph-16-00322-s001.zip › Table S1.pdf]

Table S1. Excluded studies, reason for exclusion and references

Author, year	Study design	Reason for exclusion
Akesson 1999	Cross sectional and prospective cohort	Low physical load (dental professions), only symptoms as outcome. Focus on disorders of neck and upper extremities, physically low workload, no quantitative data of exposure.
Andrianakos 2006	Cross sectional prevalence	No physical workload
Bieleman 2010	Cross sectional	No physical workload
Cho 2015	Cross sectional	No physical workload
Cleveland 2013	Cross sectional	No physical workload
Cooper 1998	Case control	No physical workload.<br>Occupational aspects: s. Coggon 1998
Cumming 1993	Case control	No physical workload
Cunningham 1992	Case control	Inadequate information to allow fair assessment of quality. Poor design, only farmer as job title.
Dahaghin 2005	Cross sectional	No physical workload
DeZwart 1997	Cohort	Only musculoskeletal symptoms as outcome.<br>No quantitative data of exposure, no comparison between exposed and not exposed.
Drawer 2001	Cross sectional	Cases ascertained by self-reported prior coxarthrosis diagnosis. No physical workload.
Goekoop 2011	Cross sectional	Not relevant. The study population are very old people (>85 years), who has very good functions. Strong selection bias!
Gomez 2003	Cross sectional	Only self-reported joint pain as outcome
Gouttebarge 2015	Review	Focus on former athletes
Holmberg 2002	Case control	Only hip pain or surgery as outcome, no exposure data.
Jacobsson 1987	Cross sectional	Not relevant (no risk assessment).<br>Weaknesses. Inadequate exposure definition, likelihood of recall bias, lack of consideration of potential confounders, and cross-sectional design.

Author, year	Study design	Reason for exclusion
Jacobsen 2004b	Cohort	Not relevant, study only among subjects without hip osteoarthritis. Joint spaces were measured, but criteria for diagnosing coxarthrosis were not specified.<br>See Jacobsen 2004a (included in this analysis).
Jacobsen 2005a	Cross sectional	See Jacobsen 2004a (included in this analysis).
Jacobsen 2005b	Case control	Not relevant, Patients with hip dysplasia, not hip osteoarthritis, no exposure assessment.
Järvholm 2008	Cohort	Poor methodology. Not relevant.<br>Exposure information limited only to job title. However there is no job title for farmer.
Juhakoski 2008	Cross sectional	Only physical function as outcome.
Keskimäki 1996	Cross sectional	Socioeconomic difference of surgery, no exposure data
Kettunen 2001	Cross sectional	Cases ascertained by self-reported prior coxarthrosis diagnosis. Same subject as those in Kujala 2014. No physical workload.
Kim 2014	Cross sectional	No physical workload
Kujala 1994	Cohort	Same subjects as in Kettunen 2001.<br>No physical workload.
Lau 2000	Case control	See Lau 2007 (included in this analysis).
Lawrence 1966	Case control	Outcome was rheumatoid arthritis, not coxarthrosis.<br>No physical workload.
Lemasters 1998	Cross sectional	Study on prevalence of musculoskeletal disorders, no data on coxarthrosis.
Lindberg 1984	Cross sectional	Unquantified exposures, no risk estimates
Litwic 2013	Review	Exposure not issued
Marti 1989	Cohort	Observational study. Exposures due to sports (recreational or professional) rather than “standard” occupations. No physical workload.
Olsen 1994	Case control	See Vingard 1991b (included in this analysis).
Rapala 2015	Cross sectional	Not relevant, only patients with osteoarthritis. No healthy control person.
Reijman 2005	Cohort	Study focused on progression from mild to severe coxarthrosis, not etiology of coxarthrosis. No risk assessment.
Rossignol 2003	Cross sectional	Not relevant, no exposure assessment.<br>Weaknesses. Lack of precise diagnostic criteria. Lack of information on potential confounders other than age and gender. Lack of quantitative exposure estimates.

Author, year	Study design	Reason for exclusion
Rossignol 2005	Cross sectional	Not relevant, no diagnosis information? Weaknesses. Use of prevalent cases. Lack of details on hip OA diagnosis. Limited, qualitative exposure definitions.
Sandmark 2000	Cross sectional	Not relevant for topic. Cases ascertained by self-reported prior coxarthrosis diagnosis.
Schmitt 2004	Cross sectional	Exposures due to sports (recreational or professional) rather than “standard” occupations, small sample size, no exposure assessment.
Schmitt 2006	Cross sectional	Exposures due to sports (recreational or professional) rather than “standard” occupations; small sample size, no exposure assessment.
Seok 2017	Cross sectional	Not relevant, no exposure information.
Shepard 2003	Cross sectional	Cases ascertained by self-reported prior coxarthrosis diagnosis, no exposure assessment, injury as confounder?
Sobti 1997	Cross sectional	Study focus on pain syndromes and stiffness.
Teichtahl 2015	Cohort	Not relevant, outcome of study is not hip osteoarthritis.
Teitz 1998	Cohort	Joint spaces were measured, but criteria for diagnosing coxarthrosis were not specified, no exposure assessment.
Tepper 1993	Case control	Factors associated with hip osteoarthritis, but no data on occupation.
Toren 2002	Cross sectional	No data on coxarthrosis, just quantification of time of tractor driving. Focus on vibration as exposure.
Typpö 1995	Cross sectional	Unclear exposure categories. Lack of risk estimates.
Van Dijk 1995	Cross sectional	No exposure data, joint disease in female ballet dancers
Vingard 1998	Case control	See Vingard 1997b: included in this analysis
Vingard 1993	Case control	Exposures due to sports (recreational or professional) rather than “standard” occupations,<br>no occupational exposure.<br>Same study group like Vingard 1991.
Xiao 2013	Cross sectional	No data on coxarthrosis.

This list is not exhaustive.

References of excluded studies

Akesson I, Johnsson B, Rylander L, Moritz U, Skerfving S. Musculoskeletal disorders among female dental personnel--clinical examination and a 5-year follow-up study of symptoms. International archives of occupational and environmental health. 1999;72(6):395-403. https://www.ncbi.nlm.nih.gov/pubmed/10473839

Andrianakos AA, Kontelis LK, Karamitsos DG, Aslanidis SI, Georgountzos AI, Kaziolas GO, et al. Prevalence of symptomatic knee, hand, and hip osteoarthritis in Greece. The ESORDIG study. J Rheumatol. 2006;33(12):2507-13. https://www.ncbi.nlm.nih.gov/pubmed/17143985

Bieleman HJ, van Ittersum MW, Groothoff JW, Oostveen JC, Oosterveld FG, van der Schans CP, et al. Functional capacity of people with early osteoarthritis: a comparison between subjects from the cohort hip and cohort knee (CHECK) and healthy ageing workers. International archives of occupational and environmental health. 2010;83(8):913-21. https://www.ncbi.nlm.nih.gov/pubmed/20490537

Cho HJ, Morey V, Kang JY, Kim KW, Kim TK. Prevalence and Risk Factors of Spine, Shoulder, Hand, Hip, and Knee Osteoarthritis in Community-dwelling Koreans Older Than Age 65 Years. Clin Orthop Relat Res. 2015;473(10):3307-14. https://www.ncbi.nlm.nih.gov/pubmed/26162413

Cleveland RJ, Schwartz TA, Prizer LP, Randolph R, Schoster B, Renner JB, et al. Associations of educational attainment, occupation, and community poverty with hip osteoarthritis. Arthritis Care Res (Hoboken). 2013;65(6):954-61. https://www.ncbi.nlm.nih.gov/pubmed/23225374

Cooper C, Inskip H, Croft P, Campbell L, Smith G, McLaren M, et al. Individual risk factors for hip osteoarthritis: obesity, hip injury, and physical activity. Am J Epidemiol. 1998;147(6):516-22. https://www.ncbi.nlm.nih.gov/pubmed/9521177

Cumming RG, Klineberg RJ. Epidemiological study of the relation between arthritis of the hip and hip fractures. Ann Rheum Dis. 1993;52(10):707-10. https://www.ncbi.nlm.nih.gov/pubmed/8257206

Cunningham RJ. Farmer's hip. BMJ. 1992;305(6845):118-9. https://www.ncbi.nlm.nih.gov/pubmed/1638242

Dahaghin S, Bierma-Zeinstra SM, Reijman M, Pols HA, Hazes JM, Koes BW. Does hand osteoarthritis predict future hip or knee osteoarthritis? Arthritis Rheum. 2005;52(11):3520-7. https://www.ncbi.nlm.nih.gov/pubmed/16255023

de Zwart BC, Broersen JP, Frings-Dresen MH, van Dijk FJ. Repeated survey on changes in musculoskeletal complaints relative to age and work demands. Occupational and environmental medicine. 1997;54(11):793-9. https://www.ncbi.nlm.nih.gov/pubmed/9538351

Drawer S, Fuller CW. Propensity for osteoarthritis and lower limb joint pain in retired professional soccer players. Br J Sports Med. 2001;35(6):402-8. https://www.ncbi.nlm.nih.gov/pubmed/11726474

Goekoop RJ, Kloppenburg M, Kroon HM, Dirkse LE, Huizinga TW, Westendorp RG, et al. Determinants of absence of osteoarthritis in old age. Scand J Rheumatol. 2011;40(1):68-73. https://www.ncbi.nlm.nih.gov/pubmed/20919944

Gomez MI, Hwang S, Stark AD, May JJ, Hallman EM, Pantea CI. An analysis of self-reported joint pain among New York farmers. J Agric Saf Health. 2003;9(2):143-57. https://www.ncbi.nlm.nih.gov/pubmed/12827860

Gouttebarge V, Inklaar H, Backx F, Kerkhoffs G. Prevalence of osteoarthritis in former elite athletes: a systematic overview of the recent literature. Rheumatol Int. 2015;35(3):405-18. https://www.ncbi.nlm.nih.gov/pubmed/25037899

Holmberg S, Stiernstrom EL, Thelin A, Svardsudd K. Musculoskeletal symptoms among farmers and non-farmers: a population-based study. Int J Occup Environ Health. 2002;8(4):339-45. https://www.ncbi.nlm.nih.gov/pubmed/12412852

Jacobsen S, Sonne-Holm S, Soballe K, Gebuhr P, Lund B. Factors influencing hip joint space in asymptomatic subjects. A survey of 4151 subjects of the Copenhagen City Heart Study: the Osteoarthritis Substudy. Osteoarthritis Cartilage. 2004b;12(9):698-703. https://www.ncbi.nlm.nih.gov/pubmed/15325635

Jacobsen S, Sonne-Holm S, Soballe K, Gebuhr P, Lund B. Hip dysplasia and osteoarthrosis: a survey of 4151 subjects from the Osteoarthrosis Substudy of the Copenhagen City Heart Study. Acta Orthop. 2005a;76(2):149-58. https://www.ncbi.nlm.nih.gov/pubmed/16097538

Jacobsen S, Sonne-Holm S, Soballe K, Gebuhr P, Lund B. Joint space width in dysplasia of the hip: a case-control study of 81 adults followed for ten years. J Bone Joint Surg Br. 2005b;87(4):471-7. https://www.ncbi.nlm.nih.gov/pubmed/15795195

Jacobsson B, Dalen N, Tjornstrand B. Coxarthrosis and labour. Int Orthop. 1987;11(4):311-3. https://www.ncbi.nlm.nih.gov/pubmed/3440647

Järvholm B, From C, Lewold S, Malchau H, Vingard E. Incidence of surgically treated osteoarthritis in the hip and knee in male construction workers. Occupational and environmental medicine. 2008;65(4):275-8. https://www.ncbi.nlm.nih.gov/pubmed/17928390

Juhakoski R, Tenhonen S, Anttonen T, Kauppinen T, Arokoski JP. Factors affecting self-reported pain and physical function in patients with hip osteoarthritis. Arch Phys Med Rehabil. 2008;89(6):1066-73. https://www.ncbi.nlm.nih.gov/pubmed/18503801

Keskimäki I, Salinto M, Aro S. Private medicine and socioeconomic differences in the rates of common surgical procedures in Finland. Health Policy. 1996;36(3):245-59. https://www.ncbi.nlm.nih.gov/pubmed/10172662

Kettunen JA, Kujala UM, Kaprio J, Koskenvuo M, Sarna S. Lower-limb function among former elite male athletes. Am J Sports Med. 2001;29(1):2-8. https://www.ncbi.nlm.nih.gov/pubmed/11206251

Kim C, Linsenmeyer KD, Vlad SC, Guermazi A, Clancy MM, Niu J, et al. Prevalence of radiographic and symptomatic hip osteoarthritis in an urban United States community: the Framingham osteoarthritis study. Arthritis Rheumatol. 2014;66(11):3013-7. https://www.ncbi.nlm.nih.gov/pubmed/25103598

Kujala UM, Kaprio J, Sarna S. Osteoarthritis of weight bearing joints of lower limbs in former elite male athletes. BMJ. 1994;308(6923):231-4. https://www.ncbi.nlm.nih.gov/pubmed/8111258

Lau EC, Cooper C, Lam D, Chan VN, Tsang KK, Sham A. Factors associated with osteoarthritis of the hip and knee in Hong Kong Chinese: obesity, joint injury, and occupational activities. Am J Epidemiol. 2000;152(9):855-62. https://www.ncbi.nlm.nih.gov/pubmed/11085397

Lawrence JS, Molyneux MK, Dingwall-Fordyce I. Rheumatism in foundry workers. Br J Ind Med. 1966;23(1):42-52. https://www.ncbi.nlm.nih.gov/pubmed/5295324

Lemasters GK, Atterbury MR, Booth-Jones AD, Bhattacharya A, Ollila-Glenn N, Forrester C, et al. Prevalence of work related musculoskeletal disorders in active union carpenters. Occupational and environmental medicine. 1998;55(6):421-7. https://www.ncbi.nlm.nih.gov/pubmed/9764103

Lindberg H, Danielsson LG. The relation between labor and coxarthrosis. Clin Orthop Relat Res. 1984(191):159-61. https://www.ncbi.nlm.nih.gov/pubmed/6499306

Litwic A, Edwards MH, Dennison EM, Cooper C. Epidemiology and burden of osteoarthritis. Br Med Bull. 2013;105:185-99. https://www.ncbi.nlm.nih.gov/pubmed/23337796

Marti B, Knobloch M, Tschopp A, Jucker A, Howald H. Is excessive running predictive of degenerative hip disease? Controlled study of former elite athletes. BMJ. 1989;299(6691):91-3. https://www.ncbi.nlm.nih.gov/pubmed/2504343

Olsen O, Vingard E, Koster M, Alfredsson L. Etiologic fractions for physical work load, sports and overweight in the occurrence of coxarthrosis. Scand J Work Environ Health. 1994;20(3):184-8. https://www.ncbi.nlm.nih.gov/pubmed/7973490

Rapala K, Truszczynska A, Tarnowski A. Total hip arthroplasty in the treatment of degenerative disorders in rural and urban patients - A retrospective, randomised and controlled study. Annals of agricultural and environmental medicine : AAEM. 2015;22(1):102-5. https://www.ncbi.nlm.nih.gov/pubmed/25780837

Reijman M, Hazes JM, Pols HA, Bernsen RM, Koes BW, Bierma-Zeinstra SM. Role of radiography in predicting progression of osteoarthritis of the hip: prospective cohort study. BMJ. 2005;330(7501):1183. https://www.ncbi.nlm.nih.gov/pubmed/15894555

Rossignol M, Leclerc A, Allaert FA, Rozenberg S, Valat JP, Avouac B, et al. Primary osteoarthritis of hip, knee, and hand in relation to occupational exposure. Occupational and environmental medicine. 2005;62(11):772-7. https://www.ncbi.nlm.nih.gov/pubmed/16234403

Rossignol M, Leclerc A, Hilliquin P, Allaert FA, Rozenberg S, Valat JP, et al. Primary osteoarthritis and occupations: a national cross sectional survey of 10 412 symptomatic patients. Occupational and environmental medicine. 2003;60(11):882-6. https://www.ncbi.nlm.nih.gov/pubmed/14573720

Sandmark H. Musculoskeletal dysfunction in physical education teachers. Occupational and environmental medicine. 2000;57(10):673-7. https://www.ncbi.nlm.nih.gov/pubmed/10984339

Schmitt H, Brocai DR, Lukoschek M. High prevalence of hip arthrosis in former elite javelin throwers and high jumpers: 41 athletes examined more than 10 years after retirement from competitive sports. Acta Orthop Scand. 2004;75(1):34-9. https://www.ncbi.nlm.nih.gov/pubmed/15022803

Schmitt H, Rohs C, Schneider S, Clarius M. [Is competitive running associated with osteoarthritis of the hip or the knee?]. Orthopäde. 2006;35(10):1087-92. https://www.ncbi.nlm.nih.gov/pubmed/16932832

Seok H, Choi SJ, Yoon JH, Song GG, Won JU, Kim JH, et al. The Association between Osteoarthritis and Occupational Clusters in the Korean Population: A Nationwide Study. PloS one. 2017;12(1):e0170229. https://www.ncbi.nlm.nih.gov/pubmed/28099527

Shepard GJ, Banks AJ, Ryan WG. Ex-professional association footballers have an increased prevalence of osteoarthritis of the hip compared with age matched controls despite not having sustained notable hip injuries. Br J Sports Med. 2003;37(1):80-1. https://www.ncbi.nlm.nih.gov/pubmed/12547750

Sobti A, Cooper C, Inskip H, Searle S, Coggon D. Occupational physical activity and long-term risk of musculoskeletal symptoms: a national survey of post office pensioners. Am J Ind Med. 1997;32(1):76-83. https://www.ncbi.nlm.nih.gov/pubmed/9131214

Teichtahl AJ, Smith S, Wang Y, Wluka AE, O'Sullivan R, Giles GG, et al. Occupational risk factors for hip osteoarthritis are associated with early hip structural abnormalities: a 3.0 T magnetic resonance imaging study of community-based adults. Arthritis Res Ther. 2015;17:19. https://www.ncbi.nlm.nih.gov/pubmed/25627648

Teitz CC, Kilcoyne RF. Premature osteoarthrosis in professional dancers. Clin J Sport Med. 1998;8(4):255-9. https://www.ncbi.nlm.nih.gov/pubmed/9884788

Tepper S, Hochberg MC. Factors associated with hip osteoarthritis: data from the First National Health and Nutrition Examination Survey (NHANES-I). Am J Epidemiol. 1993;137(10):1081-8. https://www.ncbi.nlm.nih.gov/pubmed/8317437

Toren A, Oberg K, Lembke B, Enlund K, Rask-Andersen A. Tractor-driving hours and their relation to self-reported low-back and hip symptoms. Appl Ergon. 2002;33(2):139-46. https://www.ncbi.nlm.nih.gov/pubmed/12009120

Typpö T. Osteoarthritis of the hip. Radiologic findings and etiology. Ann Chir Gynaecol Suppl. 1985;201:1-38. https://www.ncbi.nlm.nih.gov/pubmed/3911867

van Dijk CN, Lim LS, Poortman A, Strubbe EH, Marti RK. Degenerative joint disease in female ballet dancers. Am J Sports Med. 1995;23(3):295-300. https://www.ncbi.nlm.nih.gov/pubmed/7661255

Vingard E, Alfredsson L, Goldie I, Hogstedt C. Sports and osteoarthrosis of the hip. An epidemiologic study. Am J Sports Med. 1993;21(2):195-200. https://www.ncbi.nlm.nih.gov/pubmed/8465912

Vingard E, Alfredsson L, Malchau H. Osteoarthrosis of the hip in women and its relationship to physical load from sports activities. Am J Sports Med. 1998;26(1):78-82. https://www.ncbi.nlm.nih.gov/pubmed/9474406

Xiao H, McCurdy SA, Stoecklin-Marois MT, Li CS, Schenker MB. Agricultural work and chronic musculoskeletal pain among Latino farm workers: the MICASA study. Am J Ind Med. 2013;56(2):216-25. https://www.ncbi.nlm.nih.gov/pubmed/23023585
